# Alkylcysteine Sulfoxide C–S Monooxygenase Uses a Flavin-Dependent Pummerer Rearrangement

**DOI:** 10.1021/jacs.3c03545

**Published:** 2023-05-25

**Authors:** Sohan Hazra, Tadhg P. Begley

**Affiliations:** Department of Chemistry, Texas A&M University, College Station, Texas 77843, United States

## Abstract

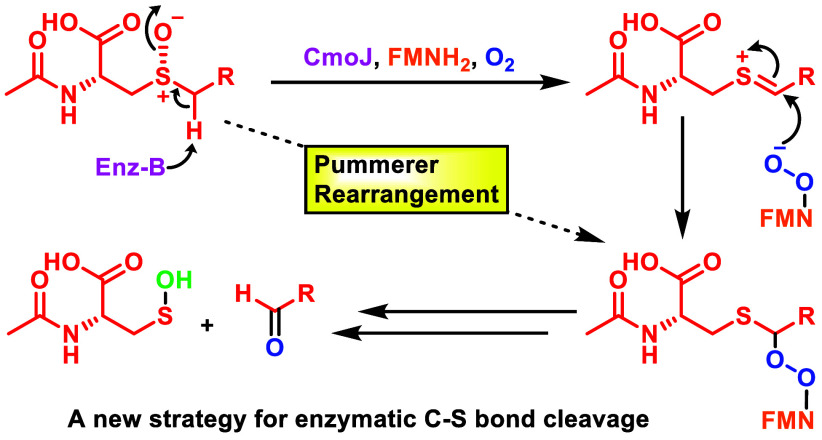

Flavoenzymes are highly versatile and participate in the catalysis of a wide range of reactions, including key reactions in the metabolism of sulfur-containing compounds. S-Alkyl cysteine is formed primarily by the degradation of S-alkyl glutathione generated during electrophile detoxification. A recently discovered S-alkyl cysteine salvage pathway uses two flavoenzymes (CmoO and CmoJ) to dealkylate this metabolite in soil bacteria. CmoO catalyzes a stereospecific sulfoxidation, and CmoJ catalyzes the cleavage of one of the sulfoxide C–S bonds in a new reaction of unknown mechanism. In this paper, we investigate the mechanism of CmoJ. We provide experimental evidence that eliminates carbanion and radical intermediates and conclude that the reaction proceeds via an unprecedented enzyme-mediated modified Pummerer rearrangement. The elucidation of the mechanism of CmoJ adds a new motif to the flavoenzymology of sulfur-containing natural products and demonstrates a new strategy for the enzyme-catalyzed cleavage of C–S bonds.

Sulfur-containing metabolites are found throughout biological systems, and a diverse array of enzymes has evolved to catalyze the cleavage of carbon–sulfur bonds. Among these enzymes are well-known examples such as PLP-dependent C–S lyases,^1,2^ redox-neutral S-(2-succino)cysteine lyase,^3^ radical SAM enzymes,^4^ SAM-dependent methyltransferases,^5^ cysteine desulfidases,^6^ and thioesterases.^7^ These enzymes use a variety of well-characterized catalytic strategies. Flavoenzymes also play an important role in C–S bond cleavage reactions in the metabolism of diverse compounds. These include S-prenylcysteine,^8^ alkanesulfonates (SsuD and MsuD),^9^ dimethyl sulfide (DmoA),^10^ dimethyl sulfone (SfnG),^11^ dibenzothiophene sulfone (DszA),^12^ and N-acetyl-S-(2-succino)cysteine (YxeK).^13^ Some flavoenzymes, such as S-prenylcysteine lyase, oxidize the C–S bond to a thionium ion, which then undergoes hydrolysis. Others, such as DszA and YxeK, are proposed to use a nucleophilic flavin peroxide to oxidatively cleave the C–S bond.^12,13^ CmoJ, which is a sequence homologue of YxeK and DszA, is involved in salvaging cysteine from S-alkylated cysteines.^14,15^

S-Alkylated cysteine is formed in the dealkylation of DNA,^16^ and in the salvage of cysteine from alkylated glutathione and related low molecular weight thiols involved in the detoxification of environmental alkylating agents.^17^ The recently discovered cysteine salvage pathway^15^ shown in Figure 1(14) is widely distributed in soil bacteria. In this pathway, S-alkylated cysteine is acetylated to **2** and oxidized to give sulfoxide **3**. CmoJ then catalyzes the cleavage of this sulfoxide to give sulfenic acid **4**. Reduction of **4** followed by deacetylation completes the salvage pathway.^14^ The C–S bond cleavage reaction shown in the conversion of **3** to **4** is an unprecedented flavoenzyme-mediated C–S bond cleavage. This communication describes an experimental strategy to elucidate the mechanism of this reaction.

**Figure 1:**
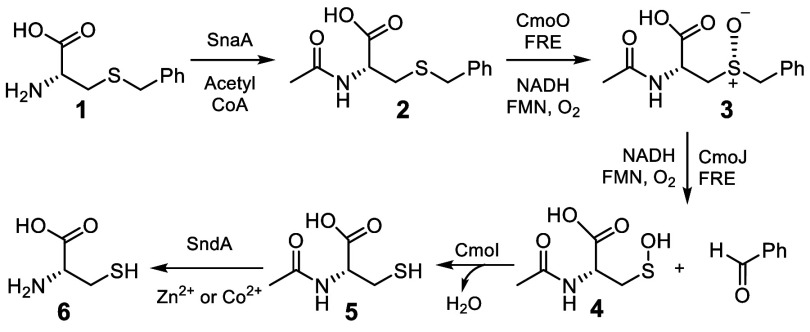
S-Benzyl cysteine salvage pathway in *B. subtilis* (FRE: Flavin reductase).

In mechanism I, deprotonation of sulfoxide **3** followed by flavin C4a-hydroperoxide (**8**) mediated hydroxylation forms **9** (Figure 2).^14^ This then undergoes a facile elimination to give sulfenic acid **4** and benzaldehyde. To test this mechanism, the CmoJ reaction was performed in D_2_O buffer under anaerobic conditions. In the absence of oxygen, flavin C4a-hydroperoxide (**8**) would not be formed, thus blocking the hydroxylation reaction. Under these conditions, prolonged incubation of the enzyme and the substrate should provide a sensitive assay for H/D exchange at Cα of the sulfoxide. In the event, LC-MS analysis of the reaction mixture after 2 h of incubation at 37 °C demonstrated that no exchange had occurred (Figure S5). Failure of mechanism I was not entirely unanticipated because the p*K*_a_ of the benzyl sulfoxide (p*K*_a_ ∼ 27)^18^ is well outside the window of accessible deprotonations in biological systems. When this experiment was repeated with the more acidic sulfone **10** (p*K*_a_ ∼ 20),^19^ exchange was detected confirming the presence of an active site base close to the Cα of the sulfoxide **3** (Figure S6).

**Figure 2:**
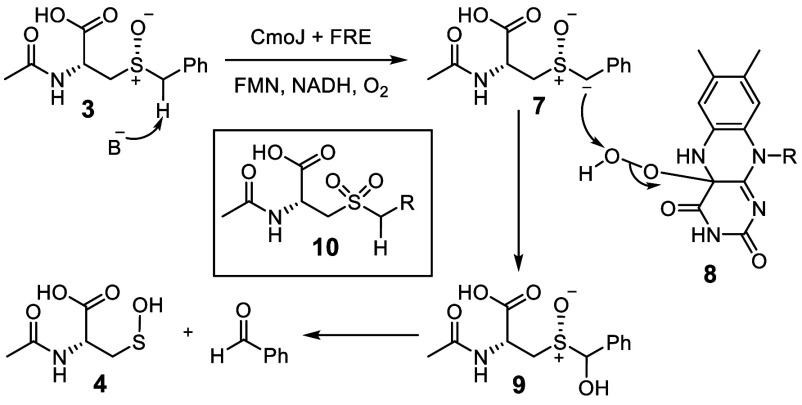
Mechanism I for the CmoJ-catalyzed C–S bond cleavage reaction. Inset shows the sulfone analog **10**.

Enzymes have evolved three widely used strategies for the catalysis of difficult deprotonation reactions: (1) concerted deprotonation where the proton abstraction is coupled to other chemical steps in the reaction (e.g., deprotonation of a thioester where deprotonation is concerted with carbonyl group protonation);^20−22^ (2) stabilization of the carbanion intermediate by extensive delocalization (e.g., PLP-dependent enzymes rely on the extensive delocalization of the resulting carbanion to enable deprotonation of α-protons in amino acids);^23,24^ (3) replacement of the deprotonation by a radical-mediated hydrogen atom abstraction (e.g., radical-mediated epimerization during the biosynthesis of hygromycin and neomycin).^25,26^ We next turned our focus on evaluating the possibility of a radical-based mechanism for the CmoJ-catalyzed reaction.

**Figure 3:**
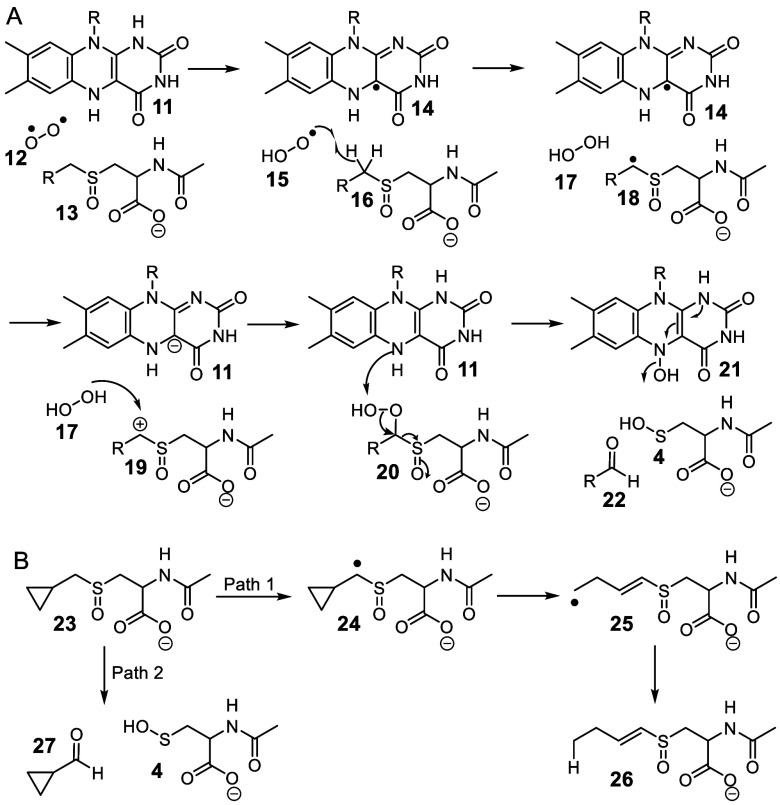
(A) Proposed radical mechanism for the CmoJ-catalyzed reaction with N-acetyl-S-alkyl-l-cysteine sulfoxide **13** (Mechanism II). (B) Proposed cyclopropyl ring-opening (Path 1) and observed products (Path 2).

A radical mechanism for CmoJ, in which flavin-generated superoxide abstracts a hydrogen atom from the substrate, is shown in Figure 3A. In this mechanism, superoxide **15**, generated by the reduction of oxygen by reduced flavin **11**, abstracts a hydrogen atom from the substrate to give radical **18**. Electron transfer from this radical to the flavin semiquinone followed by the addition of peroxide to the resulting cation gives **20** and regenerates the reduced flavin. Peroxide reduction followed by C–S bond cleavage gives **22** and **4**, and elimination of water from **21** regenerates the oxidized flavin. Substrate analog **23** was designed to test for the intermediacy of radical **24** (Figures 3B, S2, S3). With this substrate, the generation of a cyclopropyl carbinyl radical would result in rapid ring opening (rate ∼10^7^s^–1^)^27^ to give **26**, while nonradical mechanisms would give the cyclopropyl carboxaldehyde **27**. In the event, LC-MS analysis of the reaction of **23** with CmoJ followed by derivatization with dansyl hydrazine demonstrated the formation of cyclopropane carboxaldehyde **27** (Figure S9). The ring-opened vinyl sulfoxide **26** was not detected after derivatization with thiophenol (Figure S10).^28^ In addition, the CmoJ reaction with **23** followed Michaelis–Menten kinetics with no indication of radical-mediated enzyme inactivation (Figures S7, S8). These experiments suggest that the CmoJ-catalyzed C–S bond cleavage reaction does not proceed via a radical intermediate as proposed in Figure 3A.

**Figure 4:**
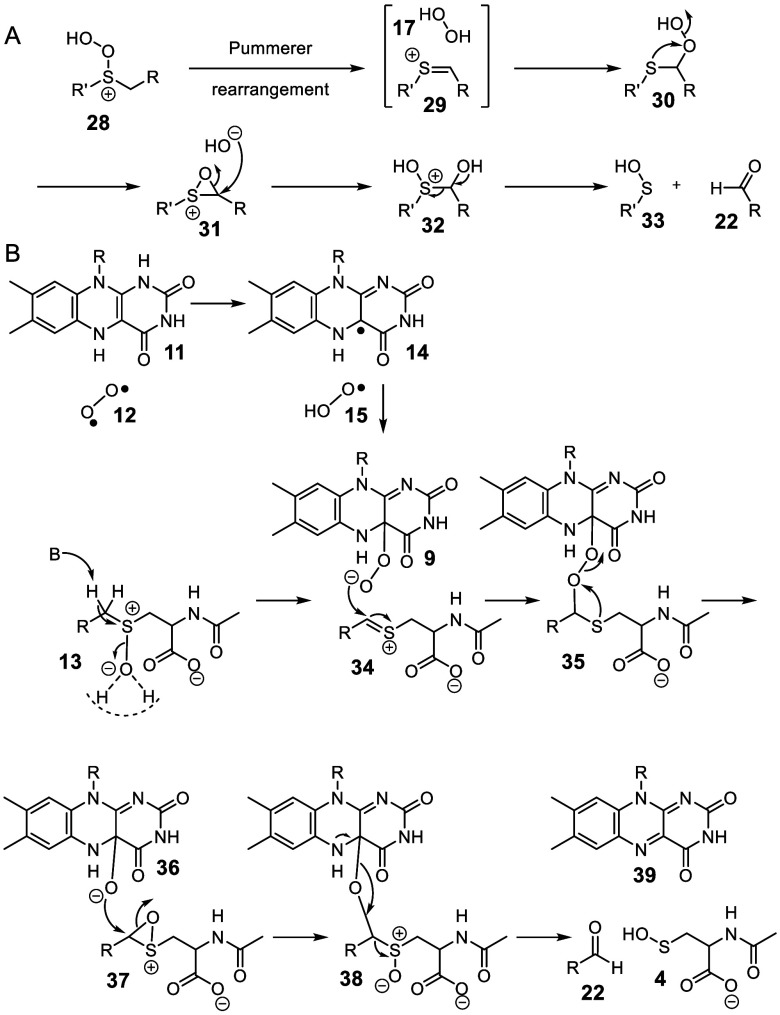
(A) Clennan’s mechanism for the C–S bond cleavage in peroxythioethers. (B) Mechanism III for the CmoJ-catalyzed reaction.

Previous mechanistic studies on the photooxidation of thioethers suggested a third possible mechanism.^29^ These studies demonstrated that thioether photooxidation proceeds via a persulfoxide intermediate **28** which then undergoes a Pummerer rearrangement, via intermediate **29**, to give α-peroxythioether **30**. Intramolecular oxidation of the sulfur via oxathiiranium **31** gives **32** which then fragments to give sulfenic acid **33** and aldehyde **22** (Figure 4A). This chemistry has a striking resemblance to the CmoJ-catalyzed reaction and suggests the mechanistic proposal shown in Figure 4B. Acid-catalyzed elimination of water from sulfoxide **13** generates the thionium ion **34**.^30^ Flavin C4a-hydroperoxide addition to **34** followed by peroxide fragmentation gives oxathiiranium ion **37**. Nucleophilic attack by the flavin C4a-hydroxide anion **36** opens the oxathiiranium ring. Elimination of the flavin adduct triggers the cleavage of the C–S bond to form the aldehyde **22** and sulfenic acid **4**.

Mechanism III is consistent with our previous observation that the aldehyde oxygen is derived from molecular oxygen^14^ and predicts that the oxygen of the sulfenic acid product **4** is also derived from molecular oxygen and not from the sulfoxide substrate **13**. Analysis of the source of the sulfenic acid oxygen is complex because sulfenic acids are highly reactive compounds with both nucleophilic and electrophilic reactivity and undergo rapid dimerization and reduction reactions.^31^ To facilitate this analysis, the sulfenic acid product was trapped in situ with phenyl vinyl sulfone **40** (PVSu, Figures 5A, and S11, S12).^32^ Substrate sulfoxide ^18^O-**3**, prepared using CmoO and ^18^O_2_ (Figure S13), was incubated with CmoJ, and the resulting sulfenic acid was trapped in situ with PVSu. MS analysis demonstrated almost complete (>90%) loss of the ^18^O label consistent with mechanism III (Figure S14 A).

**Figure 5:**
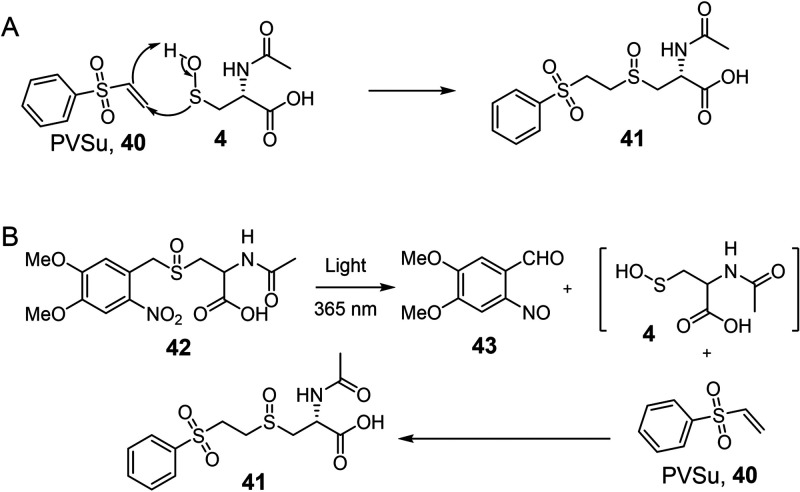
(A) The sulfenic acid trapping strategy, with phenyl vinyl sulfone (PVSu), used in the oxygen labeling experiments. PVSu was found not to inhibit CmoJ. (B) The strategy for the synthesis and trapping of N-acetylcysteine sulfenic acid using the photocaged precursor **42**.

To further establish the origin of the sulfenic acid oxygen (water or O_2_), the CmoJ reaction was run in H_2_^18^O/H_2_O (2:1)/^16^O_2_. MS analysis showed that 87% of the available ^18^O was incorporated in the trapped sulfenic acid demonstrating that the sulfenic acid oxygen was derived from the buffer, and not from molecular oxygen (Figures S14B–D, S4). Control experiments demonstrated that the substrate sulfoxide **3** and the trapped product **41** did not exchange substrate ^16^O with ^18^O from the buffer (Figure S15).

The literature on the exchange reactivity of sulfenic acids with water is sparse. While sterically protected sulfenic acids do not exchange,^33^ unhindered sulfenic acids have not been adequately studied. Therefore, the experiments above do not eliminate the possibility that initially formed sulfenic acid exchanges with water before or during the trapping reaction. To evaluate the extent of this exchange we needed access to a clean synthetic sample of N-acetylcysteine sulfenic acid **4**.

Our strategy for the synthesis and trapping of N-acetylcysteine sulfenic acid, compatible with the aqueous CmoJ assay conditions, is shown in Figure 5. This strategy involved protecting the sulfenic acid with a photoremovable protecting group (Figure S16).^34^ Irradiation of **42**, in the CmoJ reaction buffer, at 365 nm gave N-acetylcysteine sulfenic acid **4** which was successfully trapped with 10-fold excess PVSu (Figures 5B, S17A–B). When the same reaction was carried out in ^18^O-buffer, labeled oxygen incorporation into **41** was not observed at high PVSu concentrations (10–20 times substrate, Figure S17C). However, increasing levels of exchange were detected as the concentration of PVSu was decreased (2–5 times substrate, Figure S17D–F, Table S1). This demonstrated that, depending on the exact reaction conditions, N-acetylcysteine sulfenic acid **4** can undergo exchange with water. To determine the origin of the sulfenic acid oxygen in the CmoJ product it was therefore essential to compare the extent of oxygen exchange in sulfenic acid generated under similar conditions in the enzymatic and photochemical systems. This was accomplished by running reactions in which PVSu was in excess (10 mM), substrate concentrations [**3**] = [**42**] = 1 mM, and by matching the light intensity and the enzyme concentrations to equalize the rates of N-acetylcysteine sulfenic acid production. Under these conditions, photochemically generated sulfenic acid did not exchange with the buffer containing H_2_^18^O/H_2_O (1:1). In contrast, the CmoJ-generated sulfenic acid showed high oxygen atom incorporation from the same buffer (∼80% of available ^18^O, Figure S18), demonstrating that the oxygen of the CmoJ-generated sulfenic acid was buffer derived as a consequence of the catalytic mechanism and not as an exchange artifact before or during the sulfenic acid trapping. This observation is inconsistent with proposed mechanisms I, II, and III.

**Figure 6:**
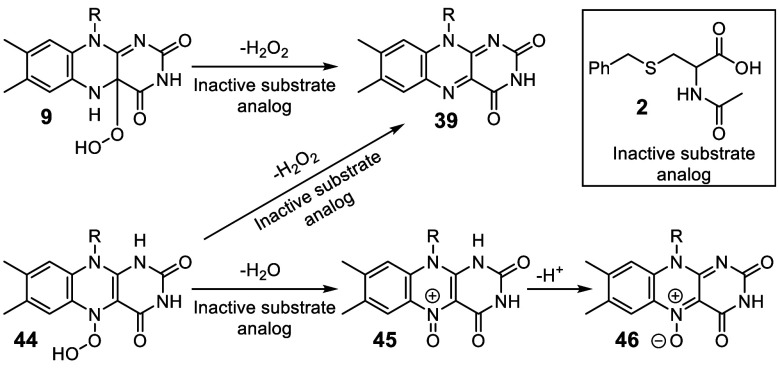
Strategy for the identification of the flavin peroxide regioisomer used in the CmoJ-catalyzed reaction.

Two regio-isomers of the flavin hydroperoxide have been previously characterized differing in the site of attachment of the peroxide to the flavin (**9** and **44**). A strategy, previously developed to identify the flavin peroxide regio-isomer in the RutA-catalyzed reaction, is shown in Figure 6.^35^ In this strategy, the enzymatic reaction is run with an inactive substrate analog **2**. Under these conditions, the C4a-hydroperoxy flavin eliminates hydrogen peroxide to give oxidized flavin **39**. In contrast, the N5-hydroperoxy flavin eliminates water to give the flavin N5-oxide **46** in addition to hydrogen peroxide elimination to give **39**. Therefore, the detection of flavin N5-oxide **46** in a flavoenzyme reaction mixture can be used as evidence for the intermediacy of the N5-hydroperoxy flavin. In the event, LC-MS analysis of the reaction mixture resulting from the aerobic incubation of CmoJ with substrate analog **2** and reduced flavin demonstrated the formation of flavin N5-oxide (Figure S19), indicating the likely involvement of N5-hydroperoxy flavin in the CmoJ-catalyzed reaction. However, we cannot yet rule out the possibility that both regio-isomers of the flavin hydroperoxide are catalytically competent.

**Figure 7:**
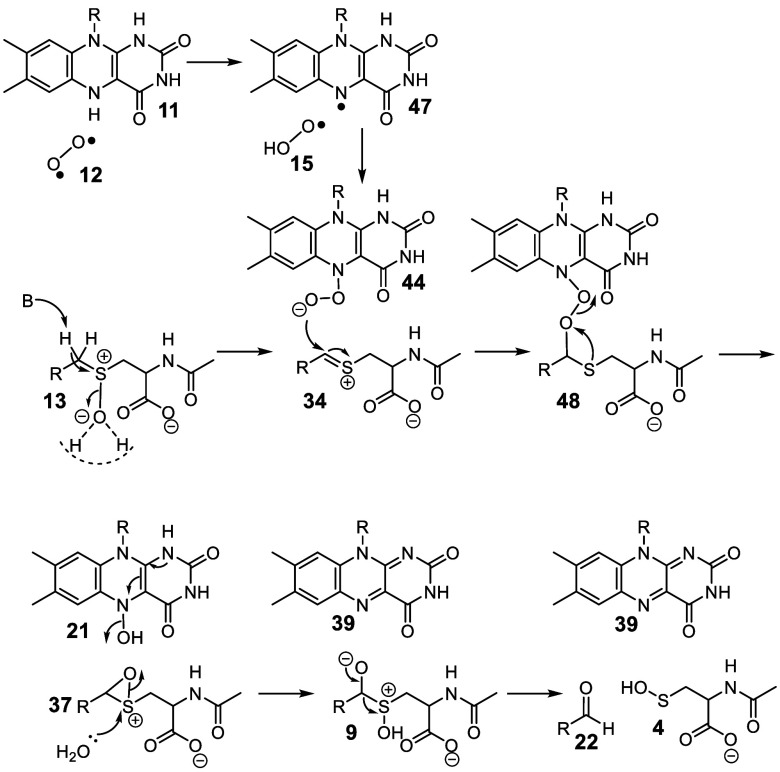
Current mechanistic proposal for the CmoJ-catalyzed reaction.

In summary, our current mechanism for the alkylcysteine sulfoxide C–S monooxygenase is shown in Figure 7. A concerted elimination of water from **13** followed by the addition of the N5 peroxy-flavin **44** to the resulting thionium ion **34** gives peroxide **48**. The regiochemistry of this addition is consistent with a large body of organic chemistry demonstrating exclusive nucleophilic addition to the carbon of thionium ions.^30,36−39^ Peroxide fragmentation gives oxathiiranium ion **37** and reduced N-5 hydroxyflavin **21**. Loss of water from the flavin gives **39**, and the addition of water from the buffer to the sulfur of the oxathiiranium ion **37** gives α-hydroxysulfoxide **9** which fragments to form the aldehyde (**22**) and the sulfenic acid (**4**) products, respectively. This proposal solves the problem of the unfavorable sulfoxide deprotonation discussed in our previous publication^14^ and is supported by oxygen labeling experiments which demonstrate that the aldehyde oxygen is derived from molecular oxygen and the sulfenic acid oxygen is derived from the buffer. The absence of CmoJ-catalyzed proton exchange between the substrate and the buffer in the anaerobic reaction (Figure S5) suggests that formation of the flavin hydroperoxide activates the enzyme for the elimination of water from the substrate **13**. In addition, the formation of the flavin N5-oxide when the CmoJ reaction is run in the presence of inhibitor **2** is consistent with the assigned regiochemistry of the peroxy flavin **44**. To the best of our knowledge, this mechanism describes the first example of an enzymatic Pummerer rearrangement^40^ as well as a new catalytic strategy for the biochemical cleavage of C–S bonds.

Glutathione or related low molecular weight thiols are widely used for the deactivation of toxic electrophiles, by S-conjugate formation. While it has been reported that such S-conjugates undergo intracellular or extracellular hydrolysis that eventually leads to the formation of cysteine S-conjugates,^17,41−43^ the fate of these cysteine derivatives was not known. Our previous report^14^ suggests the role of soil bacteria in the catabolism of such cysteine S-conjugates, by salvaging the cysteine. It had been shown that the pathway utilizes two flavoenzymes (CmoO and CmoJ) to activate and cleave the C–S bond. CmoO oxidizes the thioether to the sulfoxide specifically, while CmoJ performs an oxidative C–S cleavage. In this report, we propose the C–S bond cleavage step proceeds via an oxathiiranium intermediate **37.** This discovery adds yet another example to the growing number of new flavin catalytic motifs discovered over the past 20 years.^44−53^

## Supporting Information Available

Detailed procedures for the synthesis of all substrates and products, protein purification, enzymatic reaction conditions, and HPLC, NMR, and ESI-MS analysis of enzymatic reactions (PDF)

## References

[ref1] Lee D.; Jeong S.; Ahn J.; Ha N.-C.; Kwon A.-R. Crystal Structure of Bacterial Cystathionine Γ-Lyase in The Cysteine Biosynthesis Pathway of Staphylococcus aureus. Crystals 2019, 9 (12), 656 10.3390/cryst9120656.

[ref2] Musah R. A.; He Q.; Kubec R. Discovery and characterization of a novel lachrymatory factor synthase in Petiveria alliacea and its influence on alliinase-mediated formation of biologically active organosulfur compounds. Plant Physiol. 2009, 151 (3), 1294–1303. 10.1104/pp.109.142539. 19692535 PMC2773066

[ref3] Hillmann K. B.; Goethel M. E.; Erickson N. A.; Niehaus T. D. Identification of a S-(2-succino) cysteine breakdown pathway that uses a novel S-(2-succino) lyase. J. Biol. Chem. 2022, 298 (12), 102639 10.1016/j.jbc.2022.102639. 36309089 PMC9706529

[ref4] Shisler K. A.; Broderick J. B. Emerging themes in radical SAM chemistry. Curr. Opin. Struct. Biol. 2012, 22 (6), 701–710. 10.1016/j.sbi.2012.10.005. 23141873 PMC4083504

[ref5] Struck A. W.; Thompson M. L.; Wong L. S.; Micklefield J. S-adenosyl-methionine-dependent methyltransferases: highly versatile enzymes in biocatalysis, biosynthesis and other biotechnological applications. ChemBioChem. 2012, 13 (18), 2642–2655. 10.1002/cbic.201200556. 23180741

[ref6] Tchong S.-I.; Xu H.; White R. H. l-Cysteine Desulfidase: An [4Fe-4S] Enzyme Isolated from Methanocaldococcus jannaschii That Catalyzes the Breakdown of l-Cysteine into Pyruvate, Ammonia, and Sulfide. Biochemistry 2005, 44 (5), 1659–1670. 10.1021/bi0484769. 15683250

[ref7] Horsman M. E.; Hari T. P.; Boddy C. N. Polyketide synthase and non-ribosomal peptide synthetase thioesterase selectivity: logic gate or a victim of fate?. Nat. Prod. Rep. 2016, 33 (2), 183–202. 10.1039/C4NP00148F. 25642666

[ref8] Tschantz W. R.; Digits J. A.; Pyun H.-J.; Coates R. M.; Casey P. J. Lysosomal Prenylcysteine Lyase Is a FAD-dependent Thioether Oxidase*. J. Biol. Chem. 2001, 276 (4), 2321–2324. 10.1074/jbc.C000616200. 11078725

[ref9] Robbins J. M.; Ellis H. R. Identification of critical steps governing the two-component alkanesulfonate monooxygenase catalytic mechanism. Biochemistry 2012, 51 (32), 6378–6387. 10.1021/bi300138d. 22775358

[ref10] Boden R.; Borodina E.; Wood A. P.; Kelly D. P.; Murrell J. C.; Schafer H. Purification and Characterization of Dimethylsulfide Monooxygenase from Hyphomicrobium sulfonivorans. J. Bacteriol. 2011, 193 (5), 1250–1258. 10.1128/JB.00977-10. 21216999 PMC3067575

[ref11] Wicht D. K. The reduced flavin-dependent monooxygenase SfnG converts dimethylsulfone to methanesulfinate. Archives of biochemistry and biophysics 2016, 604, 159–166. 10.1016/j.abb.2016.07.001. 27392454

[ref12] Adak S.; Begley T. P. Dibenzothiophene catabolism proceeds via a flavin-N5-oxide intermediate. J. Am. Chem. Soc. 2016, 138 (20), 6424–6426. 10.1021/jacs.6b00583. 27120486 PMC6078383

[ref13] Matthews A.; Schönfelder J.; Lagies S.; Schleicher E.; Kammerer B.; Ellis H. R.; Stull F.; Teufel R. Bacterial flavoprotein monooxygenase YxeK salvages toxic S-(2-succino)-adducts via oxygenolytic C–S bond cleavage. FEBS J. 2022, 289 (3), 787–807. 10.1111/febs.16193. 34510734

[ref14] Hazra S.; Bhandari D. M.; Krishnamoorthy K.; Sekowska A.; Danchin A.; Begley T. P. Cysteine Dealkylation in Bacillus subtilis by a Novel Flavin-Dependent Monooxygenase. Biochemistry 2022, 61 (11), 952–955. 10.1021/acs.biochem.2c00020. 35584544

[ref15] Chan C. M.; Danchin A.; Marlière P.; Sekowska A. Paralogous metabolism: S-alkyl-cysteine degradation in Bacillus subtilis. Environ. Microbiol. 2014, 16 (1), 101–117. 10.1111/1462-2920.12210. 23944997

[ref16] Kleibl K. Molecular mechanisms of adaptive response to alkylating agents in Escherichia coli and some remarks on O6-methylguanine DNA-methyltransferase in other organisms. Mutat. Res. Rev. Mutat. Res. 2002, 512 (1), 67–84. 10.1016/S1383-5742(02)00025-X. 12220590

[ref17] Wolf A. E.; Dietz K.-J.; Schröder P. Degradation of glutathione S-conjugates by a carboxypeptidase in the plant vacuole. FEBS Lett. 1996, 384 (1), 31–34. 10.1016/0014-5793(96)00272-4. 8797797

[ref18] Bordwell F.; Matthews W. S. Equilibrium acidities of carbon acids. II. Hydrocarbon indicators, phenylacetylene, and other carbon acids in the 20–27 pK region. J. Am. Chem. Soc. 1974, 96 (4), 1214–1216. 10.1021/ja00811a040.

[ref19] Pearson R. G.; Dillon R. L. Rates of ionization of pseudo acids. 1 IV. relation between rates and equilibria. J. Am. Chem. Soc. 1953, 75 (10), 2439–2443. 10.1021/ja01106a048.

[ref20] Gerlt J. A.; Gassman P. G. An explanation for rapid enzyme-catalyzed proton abstraction from carbon acids: importance of late transition states in concerted mechanisms. J. Am. Chem. Soc. 1993, 115 (24), 11552–11568. 10.1021/ja00077a062.

[ref21] Gerlt J. A.; Gassman P. G. Understanding enzyme-catalyzed proton abstraction from carbon acids: details of stepwise mechanisms for. beta.-elimination reactions. J. Am. Chem. Soc. 1992, 114 (15), 5928–5934. 10.1021/ja00041a004.

[ref22] Sargent A. L.; Rollog M. E.; Almlöf J. E.; Gassman P. G.; Gerlt J. A. Enzyme-catalyzed enolization reactions: a theoretical study on the energetics of concerted and stepwise pathways. J. Mol. Struc.-Theochem 1996, 388, 145–159. 10.1016/S0166-1280(96)80028-5.

[ref23] Richard J. P.; Amyes T. L.; Crugeiras J.; Rios A. Pyridoxal 5′-phosphate: electrophilic catalyst extraordinaire. Curr. Opin. Chem. Biol. 2009, 13 (4), 475–483. 10.1016/j.cbpa.2009.06.023. 19640775 PMC2749917

[ref24] Major D. T.; Nam K.; Gao J. Transition State Stabilization and α-Amino Carbon Acidity in Alanine Racemase. J. Am. Chem. Soc. 2006, 128 (25), 8114–8115. 10.1021/ja062272t. 16787057

[ref25] Besandre R. A.; Chen Z.; Davis I.; Zhang J.; Ruszczycky M. W.; Liu A.; Liu H.-w. HygY is a twitch radical SAM epimerase with latent dehydrogenase activity revealed upon mutation of a single cysteine residue. J. Am. Chem. Soc. 2021, 143 (37), 15152–15158. 10.1021/jacs.1c05727. 34491039 PMC8611816

[ref26] Kudo F.; Hoshi S.; Kawashima T.; Kamachi T.; Eguchi T. Characterization of a radical S-adenosyl-L-methionine epimerase, NeoN, in the last step of neomycin B biosynthesis. J. Am. Chem. Soc. 2014, 136 (39), 13909–13915. 10.1021/ja507759f. 25230155

[ref27] Effio A.; Griller D.; Ingold K.; Beckwith A.; Serelis A. Allylcarbinyl-cyclopropylcarbinyl rearrangement. J. Am. Chem. Soc. 1980, 102 (5), 1734–1736. 10.1021/ja00525a051.

[ref28] Santa T. Derivatization reagents in liquid chromatography/electrospray ionization tandem mass spectrometry. Biomed. Chromatogr. 2011, 25 (1–2), 1–10. 10.1002/bmc.1548. 21058414

[ref29] Toutchkine A.; Aebisher D.; Clennan E. L. Substituent-Dictated Partitioning of Intermediates on the Sulfide Singlet Oxygen Reaction Surface. A New Mechanism for Oxidative C– S Bond Cleavage in α-Hydroperoxy Sulfides. J. Am. Chem. Soc. 2001, 123 (21), 4966–4973. 10.1021/ja004188y. 11457324

[ref30] Smith L. H.; Coote S. C.; Sneddon H. F.; Procter D. J. Beyond the Pummerer reaction: recent developments in thionium ion chemistry. Angew. Chem., Int. Ed. 2010, 49 (34), 5832–5844. 10.1002/anie.201000517. 20583014

[ref31] Conte M. L.; Carroll K. S. The chemistry of thiol oxidation and detection. In Oxidative stress and redox regulation; Springer: 2013; pp 1–42.

[ref32] Gupta V.; Carroll K. S. Sulfenic acid chemistry, detection and cellular lifetime. Biochim. Biophys. Acta - Gen. Subj. 2014, 1840 (2), 847–875. 10.1016/j.bbagen.2013.05.040. PMC4184475 23748139

[ref33] Goto K.; Shimada K.; Nagahama M.; Okazaki R.; Kawashima T. Reaction of stable sulfenic and selenenic acids containing a bowl-type steric protection group with a phosphine. Elucidation of the mechanism of reduction of sulfenic and selenenic acids. Chem. Lett. 2003, 32 (11), 1080–1081. 10.1246/cl.2003.1080.

[ref34] Pan J.; Carroll K. S. Light-Mediated Sulfenic Acid Generation from Photocaged Cysteine Sulfoxide. Org. Lett. 2015, 17 (24), 6014–6017. 10.1021/acs.orglett.5b02981. 26641493 PMC4699549

[ref35] Matthews A.; Saleem-Batcha R.; Sanders J. N.; Stull F.; Houk K.; Teufel R. Aminoperoxide adducts expand the catalytic repertoire of flavin monooxygenases. Nat. Chem. Biol. 2020, 16 (5), 556–563. 10.1038/s41589-020-0476-2. 32066967

[ref36] Bur S. K.; Padwa A. The Pummerer Reaction: Methodology and Strategy for the Synthesis of Heterocyclic Compounds. Chem. Rev. 2004, 104 (5), 2401–2432. 10.1021/cr020090l. 15137795

[ref37] Carreño-Montero A.; Maldonado L. A.; Chávez M. I.; Hernández-Ortega S.; Delgado G. An unexpected Pummerer rearrangement in the synthetic route to ethyl (2′-hydroxy-4′,5′-methylenedioxyphenyl)acetate: An alternative approach to 2,3-dimethylthio benzofurans. Tetrahedron Lett. 2019, 60 (48), 151282 10.1016/j.tetlet.2019.151282.

[ref38] Saha P.; Ray S. K.; Singh V. K. Copper-catalyzed Pummerer type reaction of α-thio aryl/heteroarylacetates: Synthesis of aryl/heteroaryl α-keto esters. Tetrahedron Lett. 2017, 58 (18), 1765–1769. 10.1016/j.tetlet.2017.03.069.

[ref39] Shimizu A.; Takeda K.; Mishima S.; Saito K.; Kim S.; Nokami T.; Yoshida J.-i. Generation, Characterization, and Reactions of Thionium Ions Based on the Indirect Cation Pool Method. Bull. Chem. Soc. Jpn. 2016, 89 (1), 61–66. 10.1246/bcsj.20150323.

[ref40] Feldman K. S. Modern Pummerer-type reactions. Tetrahedron 2006, 62 (21), 5003–5034. 10.1016/j.tet.2006.03.004.

[ref41] Moldèus P.; Jones D. P.; Ormstad K.; Orrenius S. Formation and metabolism of a glutathione-S-conjugate in isolated rat liver and kidney cells. Biochem. Biophys. Res. Commun. 1978, 83 (1), 195–200. 10.1016/0006-291X(78)90416-3. 697808

[ref42] Cordente A. G.; Capone D. L.; Curtin C. D. Unravelling glutathione conjugate catabolism in Saccharomyces cerevisiae: the role of glutathione/dipeptide transporters and vacuolar function in the release of volatile sulfur compounds 3-mercaptohexan-1-ol and 4-mercapto-4-methylpentan-2-one. Appl. Microbiol. Biotechnol. 2015, 99 (22), 9709–9722. 10.1007/s00253-015-6833-5. 26227410

[ref43] Bachhawat A. K.; Kaur A. Glutathione degradation. Antioxid. Redox Signal. 2017, 27 (15), 1200–1216. 10.1089/ars.2017.7136. 28537416

[ref44] Beaupre B. A.; Moran G. R. N5 is the new C4a: biochemical functionalization of reduced flavins at the N5 position. Front. Mol. Biosci. 2020, 7, 598912 10.3389/fmolb.2020.598912. 33195440 PMC7662398

[ref45] Chaiyen P. Flavoenzymes catalyzing oxidative aromatic ring-cleavage reactions. Arch. Biochem. Biophys. 2010, 493 (1), 62–70. 10.1016/j.abb.2009.08.021. 19728986

[ref46] Hamdane D.; Grosjean H.; Fontecave M. Flavin-dependent methylation of RNAs: Complex chemistry for a simple modification. J. Mol. Biol. 2016, 428 (24_Part_B), 4867–4881. 10.1016/j.jmb.2016.10.031. 27825927

[ref47] Leys D.; Scrutton N. S. Sweating the assets of flavin cofactors: new insight of chemical versatility from knowledge of structure and mechanism. Curr. Opin. Struct. Biol. 2016, 41, 19–26. 10.1016/j.sbi.2016.05.014. 27266331

[ref48] Paul C. E.; Eggerichs D.; Westphal A. H.; Tischler D.; van Berkel W. J. H. Flavoprotein monooxygenases: Versatile biocatalysts. Biotechnol. Adv. 2021, 51, 107712 10.1016/j.biotechadv.2021.107712. 33588053

[ref49] Romero E.; Gomez Castellanos J. R.; Gadda G.; Fraaije M. W.; Mattevi A. Same Substrate, Many Reactions: Oxygen Activation in Flavoenzymes. Chem. Rev. (Washington, DC, U. S.) 2018, 118 (4), 1742–1769. 10.1021/acs.chemrev.7b00650. 29323892

[ref50] Toplak M.; Matthews A.; Teufel R. The devil is in the details: The chemical basis and mechanistic versatility of flavoprotein monooxygenases. Arch. Biochem. Biophys. 2021, 698, 108732 10.1016/j.abb.2020.108732. 33358998

[ref51] Toplak M.; Teufel R. Three Rings to Rule Them All: How Versatile Flavoenzymes Orchestrate the Structural Diversification of Natural Products. Biochemistry 2022, 61 (2), 47–56. 10.1021/acs.biochem.1c00763. 34962769 PMC8772269

[ref52] Walsh C. T.; Wencewicz T. A. Flavoenzymes: Versatile catalysts in biosynthetic pathways. Nat. Prod. Rep. 2013, 30 (1), 175–200. 10.1039/C2NP20069D. 23051833 PMC3518583

[ref53] Adak S.; Jhulki I.; Begley T. P. 4.02 - New Catalytic Motifs in Flavoenzymology. In Comprehensive Natural Products III; Liu H.-W., Begley T. P., Eds.; Elsevier: 2020; pp 3–20.

