# Supplementary material for: Alkylcysteine Sulfoxide C–S Monooxygenase Uses a Flavin-Dependent Pummerer Rearrangement
Source: J Am Chem Soc. 2023 May 25;145(22):11933–8. doi: 10.1021/jacs.3c03545 (PMC10863075; doi:10.1021/jacs.3c03545)
Supplement: Supplementary file 1 — ja3c03545_si_001.pdf [file ja3c03545_si_001.pdf]

Alkylcysteine Sulfoxide C-S Monoxygenase Uses a Flavin-Dependent Pummerer Rearrangement

Sohan Hazra and Tadhg P. Begley*
Department of Chemistry, Texas A&M University, College Station, Texas 77843, United States.
*begley@chem.tamu.edu

Supporting Information

Table of contents

Materials 3
Overexpression and purification of enzymes 3
Figure S1: SDS-PAGE of purified proteins. 4
HPLC parameters 4
LC-MS parameters 4
Synthesis of N-acetyl-S-(cyclopropyl)methylcysteine 5
Figure S2: Synthesis of N-acetyl-S-(cyclopropyl)methylcysteine **50**. 5
Utilization of CmoO to synthesize substrates for CmoJ 5
Figure S3: CmoO-catalyzed sulfoxidation of S-alkylcysteines. 6
The general formula for determining the percent incorporation of a single stable isotope 7
Figure S4: Calculation of percent isotope incorporation of stable isotopes into small molecules, using LC-MS data of natural abundance and labeled species. 7
Anaerobic deuterium incorporation studies on N-acetyl-S-benzylcysteine sulfoxide **3** by CmoJ 8
Figure S5: Lack of deuterium incorporation from solvent into N-acetyl-S-benzylcysteine sulfoxide by CmoJ in the absence of oxygen. 8
Deuterium incorporation studies using N-acetyl-S-(cyclopropyl)methylcysteine sulfone **51**: ... 8
Figure S6: CmoJ-catalyzed incorporation of deuterium into N-acetyl-S-(cyclopropyl)methylcysteine sulfone **51**. 10
Derivatization of N-acetyl-L-cysteine (**5**) with 4,4'-dithiodipyridine (**53**) 11
Figure S7: Synthesis and characterization of the adduct between N-acetylcysteine and 4,4'-dithiodipyridine 11
Kinetics of CmoJ catalyzed reaction with N-acetyl-S-(cyclopropyl)methylcysteine sulfoxide 12
Figure S8: Michaelis-Menten kinetics of the CmoJ catalyzed reaction with N-acetyl-S-(cyclopropyl)methylcysteine sulfoxide **23**. 12

Detection of cyclopropane carbaldehyde **27** formation in the CmoJ-catalyzed reaction with N-acetyl-S-(cyclopropyl)methylcysteine **23** 12
Figure S9: Evidence for formation of cyclopropyl carbaldehyde **27**, in the CmoJ-catalyzed reaction with N-acetyl-S-(cyclopropyl)methylcysteine sulfoxide **23**. 13
Analysis of the CmoJ-catalyzed reaction with N-acetyl-S-(cyclopropyl)methylcysteine for vinyl sulfoxide **26** 13
Figure S10: Strategy to detect vinyl sulfoxide **26** using thiophenol trapping. 14
Sulfenic acid trapping using phenyl vinyl sulfone 14
Figure S11: Strategy for trapping the sulfenic acid product **4** in the CmoJ reaction using phenyl vinyl sulfone **40** 15
Chemoenzymatic synthesis of the adduct **41** between sulfenic acid and phenyl vinyl sulfone 15
Figure S12: Synthesis of the adduct 41 between sulfenic acid **4** and phenyl vinyl sulfone **40**. 16
Synthesis of N-acetyl-S-benzylcysteine ^{18}O-sulfoxide ([^{18}O]-**3**) using CmoO 16
Figure S13: Synthesis of N-acetyl-S-benzylcysteine ^{18}O-sulfoxide. 17
Determining the fate of the sulfoxide oxygen during the CmoJ reaction 18
Determining the source of the sulfenic acid oxygen in the CmoJ product 18
Figure S14: Determination of the origin of the sulfenic acid oxygen. 19
Model studies to probe solvent oxygen incorporation during and after adduct formation between sulfenic acid **4** and PVSu **40** 20
Figure S15: LC-MS studies on various possibilities for incorporation of solvent oxygen in adduct **41**. 20
Synthesis of photocaged N-acetylcysteine sulfenic acid precursor **42** 21
Figure S16: Synthesis of N-acetyl-S-(4,5-dimethoxy-2-nitrobenzyl)-L-cysteine sulfoxide..... 21
Photo-generation of N-acetylcysteine sulfenic acid and its *in situ* trapping with phenyl vinyl sulfone 22
Figure S17: Evaluation of oxygen exchange in photogenerated N-acetylcysteine sulfenic acid. 23
Table S1: Effect of trapping agent concentration on the exchange of N-acetylcysteine sulfenic acid **4** with buffer. 24
Evaluation of sulfenic acid oxygen exchange during the CmoJ-catalyzed reaction 24
Figure S18: Incorporation of oxygen from water into the photochemically and enzymatically produced sulfenic acid **4**. 25
Trapping of an FMN N5-peroxide intermediate in the CmoJ-catalyzed reaction 26
Figure S19: Trapping FMN N5-peroxide intermediate **46** in the CmoJ-catalyzed reaction..... 26
References 27

Materials

All chemicals were purchased from Millipore-Sigma unless specified. LB broth (Lennox formulation) was from EMD Millipore. Kanamycin was from Teknova and IPTG was obtained from Lab Scientific Inc. HPLC and LC-MS solvents were purchased from EMD and were used without further purification. His trap columns (5 ml) were obtained from GE Healthcare. Econo-Pack 10DG and Bio-spin 6 desalting columns were purchased from Bio-Rad Laboratories. Large cultures were grown and overexpressed in 2.5 L baffled ultra-yield flasks from Thomson Instrument Company. NMR tubes (3 mm and 5 mm diameter) were obtained from Wilmad-Labglass. D_2O, MeOD, and d_6-DMSO were purchased from Cambridge Isotope Laboratories Inc.

Overexpression and purification of enzymes

The genes encoding *cmoO* and *cmoJ* were cloned in the pTHT vector (a derivative of the pET28b vector with a TEV protease cleavage site after the N-terminal His-tag). The respective plasmids were transformed into *E. coli* BL21(DE3) competent cells by electroporation. Starter cultures were grown overnight in LB media containing kanamycin (40 µg/ml). 30 ml of this culture were added to 3 L LB media (2 x 1.5 L flasks) with kanamycin (40 µg/ml) and grown at 37 °C with shaking (180 rpm) till OD_{600}~0.6. The flasks were then incubated at 4 °C for ~2 hr without shaking, induced with 500 µM IPTG followed by incubation at 15 °C for ~14 hr with shaking at 180 rpm. The cells were harvested by centrifugation at 5,000 rpm for 20 min and stored in liquid nitrogen until further use. Typical yields were 9-10 g of cell pellet (wet weight) from 3 L cell culture.

For purification, the cell pellets were thawed and resuspended in 40-50 ml of lysis buffer (100 mM KPi, 150 mM NaCl, pH 7.5) at room temperature in the presence of lysozyme (6-8 mg). The suspension was stirred for ~1.5 hr on an ice bath and sonicated to lyse the cells. Cell debris was removed by centrifugation at 15,000 rpm for 20 min and the lysate was filtered using 0.22 µm filters. The filtered lysate was loaded onto a His-trap column pre-equilibrated with lysis buffer. The column was washed with 10 column volumes of wash buffer (100 mM KPi, 20 mM imidazole, 150 mM NaCl, pH 7.5). The protein was then eluted from the His-trap column with elution buffer (100 mM KPi, 250 mM imidazole, 150 mM NaCl, pH 7.5). Protein-containing fractions, identified using the Bradford reagent, were pooled and concentrated using 15 ml 10 kDa filters. The concentrated protein was buffer exchanged to 100 mM KPi, 30% glycerol, pH 7.5 using an Econo-Pac 10DG desalting column. Aliquots of 100 µL desalted enzyme in Eppendorf tubes were flash-frozen in liquid nitrogen and stored at -80 °C until further use. Protein concentration was determined by measuring the absorbance at 280 nm (A_{280}) and utilizing the extinction coefficient calculated using the ProtParam tool of the ExPASy proteomics server ($\varepsilon_{280} = 42400\ M^{-1}cm^{-1}$).

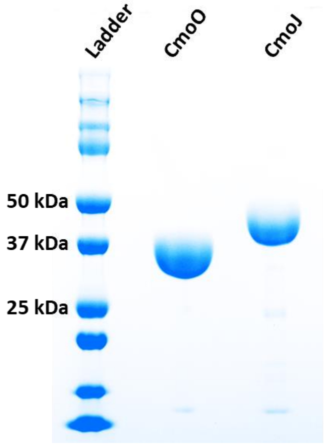

Figure S1: SDS-PAGE of purified proteins. CmoO (33 kDa) and CmoJ (49 kDa) respectively.

HPLC parameters

An Agilent 1200 or 1260 HPLC equipped with a quaternary pump and a diode array UV-Vis detector was used. Analysis was performed using either a SUPELCOSIL LC-18-T (15 cm x 4.6 mm, 3 µm particles, Sigma) or ZORBAX Eclipse XDB-C18 column (15 cm x 4.6 mm, 5 µm particles, Agilent Technologies). Data were processed using ChemStation ver. B.04.01 SP1 (Agilent technologies).

HPLC conditions:

A. Water
B. 100 mM Potassium phosphate buffer, pH 6.6
C. Methanol

HPLC method:

(Flow rate = 1 ml/min)
0 min – 100% B, 2 min – 10% A 90% B, 10 min – 45% A 15% B 40% C, 18 min – 25% A 15% B 60% C, 20 min - 25% A 15% B 60% C, 22 min - 100% B, 25 min - 100% B.

LC-MS parameters

LC-ESI-TOF-MS was performed using an Agilent 1260 HPLC system equipped with a binary pump and a 1200 series diode array detector followed by a MicroToF-Q II mass spectrometer (Bruker Daltonics) using an ESI source in negative or positive mode. Analysis was performed on an LC-18-T column (15 cm x 3 mm, 3 µm particles, Supelco). Data were processed using DataAnalysis 4.0 SP1 (Bruker Daltonics).

LC conditions:

A. 5 mM Ammonium acetate buffer, pH 6.6
B. 75% Methanol and 25% Water.

LC method: (Flow rate = 0.4 ml/min)
0 min – 100% A, 7 min – 100% A, 10 min – 80% A 20% B, 27 min – 100% B, 29 min – 100% B, 30 min – 100% A, 40 min – 100% A.

Synthesis of N-acetyl-S-(cyclopropyl)methylcysteine

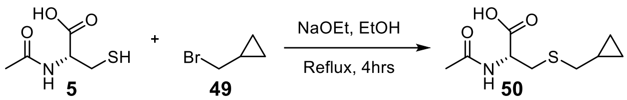

Figure S2: Synthesis of N-acetyl-S-(cyclopropyl)methylcysteine **50**.

In a 50 mL round bottom flask, commercially available N-acetylcysteine (163 mg, 1 mmol) was dissolved in 10 mL anhydrous ethanol, under an Argon atmosphere. To this was added a solution of sodium ethoxide (21% in ethanol, 0.72 mL, 2.2 eq.), and stirred for 15 minutes under an Argon atmosphere. Commercially available bromomethylcyclopropane (149 mg, 1.1 mmol) was added to the solution and stirred under reflux for 6 hrs. The solution was cooled to room temperature and 5 mL H_2O was added. The solution was evaporated to 10 mL, and ethyl acetate (50 mL), and 1M HCl (20 mL) were added and mixed thoroughly. The organic layer was separated, washed with brine (50 mL), and dried with anhydrous sodium sulfate. The solvent was evaporated, and the residue was recrystallized from a mixture of methanol, ethyl acetate, and hexane. Yield: 68% ^{1}H NMR (400 MHz, DMSO) δ 8.44 (d, *J* = 8.0 Hz, 1H), 4.62 (td, *J* = 8.3, 3.8 Hz, 1H), 3.58 (dd, *J* = 14.6, 3.8 Hz, 1H), 3.44 (dd, *J* = 14.6, 8.5 Hz, 1H), 3.06 (d, *J* = 7.1 Hz, 2H), 1.84 (s, 3H), 1.11 – 0.93 (m, 1H), 0.70 – 0.47 (m, 2H), 0.44 – 0.26 (m, 2H).

Utilization of CmoO to synthesize substrates for CmoJ

S-alkyl-N-acetylcysteine was converted to its *R*-sulfoxide using CmoO. A typical 100 µL reaction contained 200 µM CmoO, 1 µM flavin reductase (FRE), 220 µM FMN, 2-4 mM NADH, and 2 mM S-alkyl-N-acetylcysteine substrate in 100 mM KPi buffer pH 7.5, and was incubated at 37 °C for 1-6 hrs. Protein was then removed using a 10 kDa filter and 40-80 µL of the sample was analyzed by LC-MS. In most cases, substrates were further purified using HPLC and lyophilized. Otherwise, the filtered CmoO reaction mixture was used directly for the CmoJ reaction or lyophilized and later dissolved in anaerobic buffer for anaerobic reactions.

A)

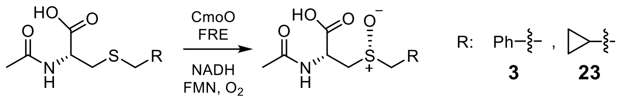

B)

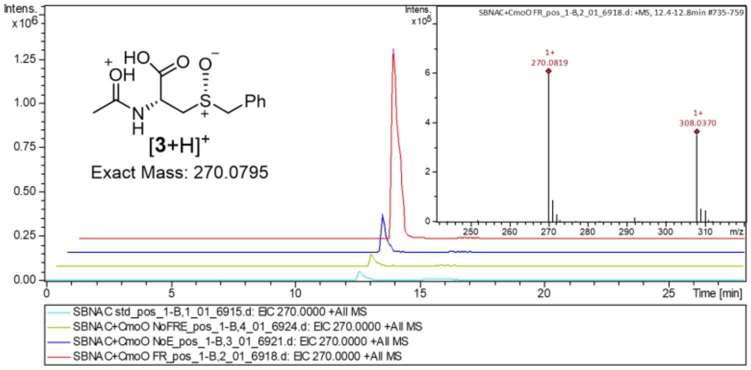

C)

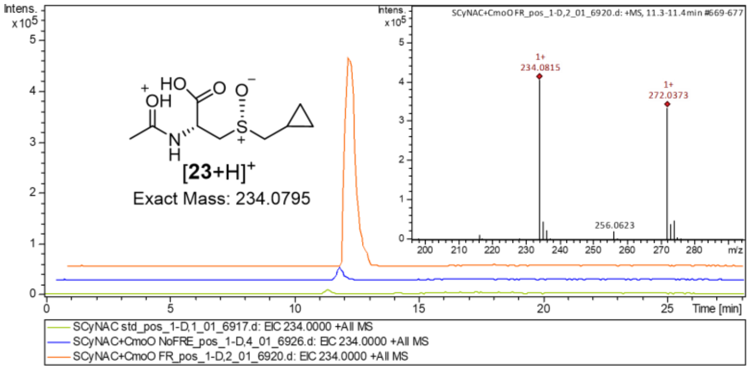

Figure S3: CmoO-catalyzed sulfoxidation of S-alkylcysteines. A) The CmoO-catalyzed sulfoxidation reaction to form substrates for CmoJ B) LC-MS analysis of the formation of N-acetyl-S-benzylcysteine sulfoxide **3**, m/z = 270 $[M+H]^+$ and 308 $[M+K]^+$ (EIC of m/z = 270 $[M+H]^+$, Red: full reaction, blue: no CmoJ, green: no FRE, cyan: N-acetyl-S-benzylcysteine standard) C) LC-MS analysis of the formation of N-acetyl-S-(cyclopropyl)methylcysteine sulfoxide **23**, m/z = 234 $[M+H]^+$ and 272 $[M+K]^+$ (EIC of m/z = 234 $[M+H]^+$, Orange: full reaction, blue: no CmoJ, green: no FRE). Small amounts of non-enzymatic oxidation can be observed due to hydrogen peroxide produced by reduced FMN reacting with oxygen.

The general formula for determining the percent incorporation of a single stable isotope

The calculation used to determine the percent incorporation of a stable isotope into a reaction product is as follows:[1]

If M is the mass of the molecular ion and M' is the mass of the next isotopologue (M' = M+1, M+2, etc.), the contribution of M' in the natural abundance molecule is xA where x is the fractional intensity of M' and A is the peak height (Figure S4A). After the label incorporation, the intensity of M' increases to B_1 (Figure S4B), the label incorporation is (B_1 – xA_1), and the exchangeable pool is (A_1 + B_1 – xA_1). Therefore:

$$\text{exchange} = \frac{100*(B_1 - xA_1)}{(A_1 + B_1 - xA_1)}.$$

If y% of the isotope source is labeled this formula becomes:

$$\%\ \text{exchange} = (100/y)*\frac{100*(B_1 - xA_1)}{(A_1 + B_1 - xA_1)}.$$

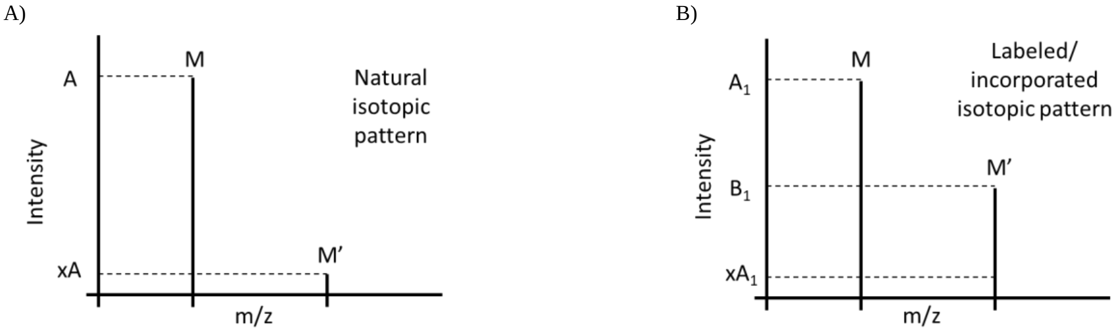

Figure S4: Calculation of percent isotope incorporation of stable isotopes into small molecules, using LC-MS data of natural abundance and labeled species. A) A typical LC-MS spectrum showing the natural isotopic distribution of a molecule. B) An LC-MS spectrum after isotope labeling showing the enhanced M' signal.

Anaerobic deuterium incorporation studies on N-acetyl-S-benzylcysteine sulfoxide 3 by CmoJ

For the anaerobic experiments, all buffers and reagents were transferred into an anaerobic chamber ($\leq$ 5 ppm O_2, COY Laboratories). FRE and CmoJ were buffer exchanged into anaerobic 100 mM KPi-D_2O pD 7.1,[2] using a Bio-spin 6 desalting column. Stock solutions of substrates (lyophilized solid as mentioned above) and cofactors were also prepared in anaerobic 100 mM KPi-D_2O pD 7.1 buffer. A 100 µL reaction mixture, containing 80 µM CmoJ, 1 µM FRE, 100 µM FMN, 1 mM NADH, and 50 µM (single turnover) and 500 µM (multiple turnovers) substrate **3** in 100 mM KPi-D_2O buffer pD 7.1, was incubated at 37 ^oC for 2 hrs. Protein was then removed using a 10 kDa filter, inside the anaerobic chamber and 80 µL of the sample was analyzed by LC-MS.

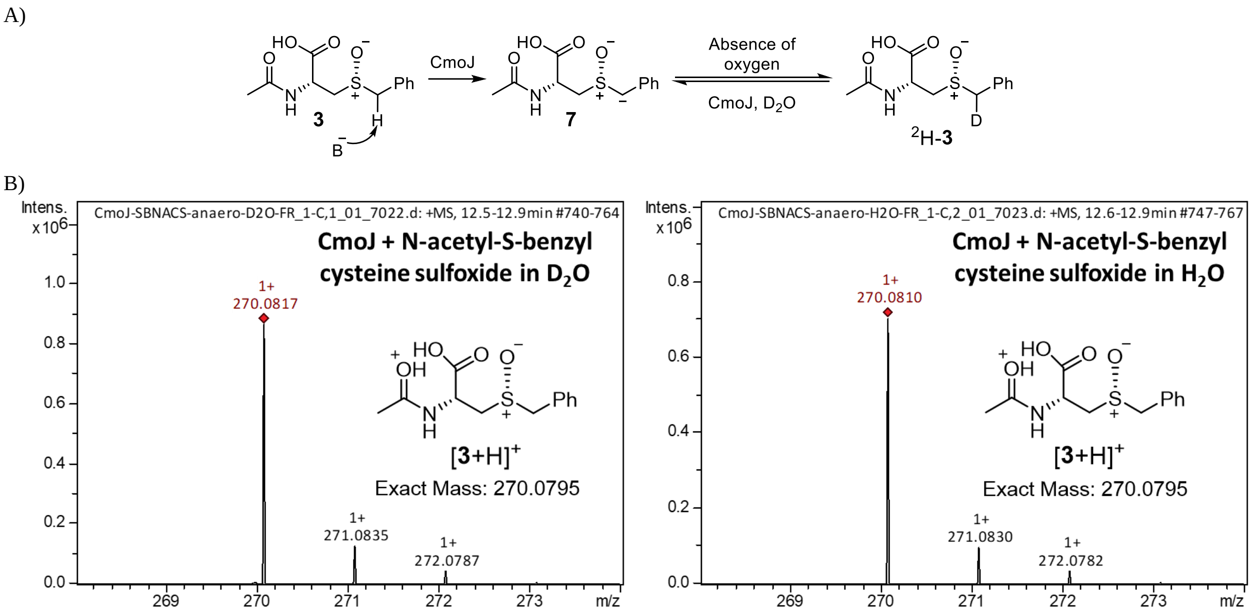

Figure S5: Lack of deuterium incorporation from solvent into N-acetyl-S-benzylcysteine sulfoxide by CmoJ in the absence of oxygen. A) Proposed deuterium incorporation into N-acetyl-S-benzylcysteine sulfoxide by CmoJ in the absence of oxygen, B) LC-MS analysis of the reaction mixture resulting from anaerobic incubation of CmoJ + N-acetyl-S-benzylcysteine sulfoxide in D_2O (left panel) and H_2O (right panel). There is no apparent increase in the m/z = 271 peak on incubation with D_2O.

Deuterium incorporation studies using N-acetyl-S-(cyclopropyl)methylcysteine sulfone 51:

The N-acetyl-S-benzylcysteine sulfone was unsuitable for the exchange study because it showed low levels of non-enzymatic exchange. The study was therefore carried out with N-acetyl-S-(cyclopropyl)methylcysteine sulfone **51** synthesized by chemical over-oxidation of the thioether **50**. The enzymatic reactions were performed under both aerobic and anaerobic conditions.

FRE and CmoJ were buffer exchanged into 100 mM KPi-D_2O pD 7.1, using a Bio-spin 6 desalting column. A 100 µL reaction mixture, containing 80 µM CmoJ, 1 µM FRE, 100 µM FMN, 1 mM NADH, and 500 µM sulfone **51** in 100 mM KPi-D_2O buffer pD 7.1, was incubated at 37 ^oC

for 2 hrs. Protein was then removed using a 10 kDa filter, and 80 µL of the sample was analyzed by LC-MS.

For the anaerobic experiments, all buffers and reagents were transferred into an anaerobic chamber ($\leq$ 5 ppm O_2, COY Laboratories). FRE and CmoJ were buffer exchanged into anaerobic 100 mM KPi-D_2O pD 7.1, using a Bio-spin 6 desalting column. Stock solutions of substrate **51** and cofactors were also prepared in anaerobic 100 mM KPi-D_2O pD 7.1. A 100 µL reaction mixture containing 80 µM CmoJ, 1 µM FRE, 100 µM FMN, 1 mM NADH, and 500 µM sulfone **51** in 100 mM KPi-D_2O buffer pD 7.1, was incubated at 37 ^{o}C for 2 hrs. Protein was then removed, inside the anaerobic chamber using a 10 kDa filter, and 80 µL of the sample was analyzed by LC-MS (Figure S6) demonstrating that **51** undergoes deuterium exchange under both aerobic and anaerobic conditions.

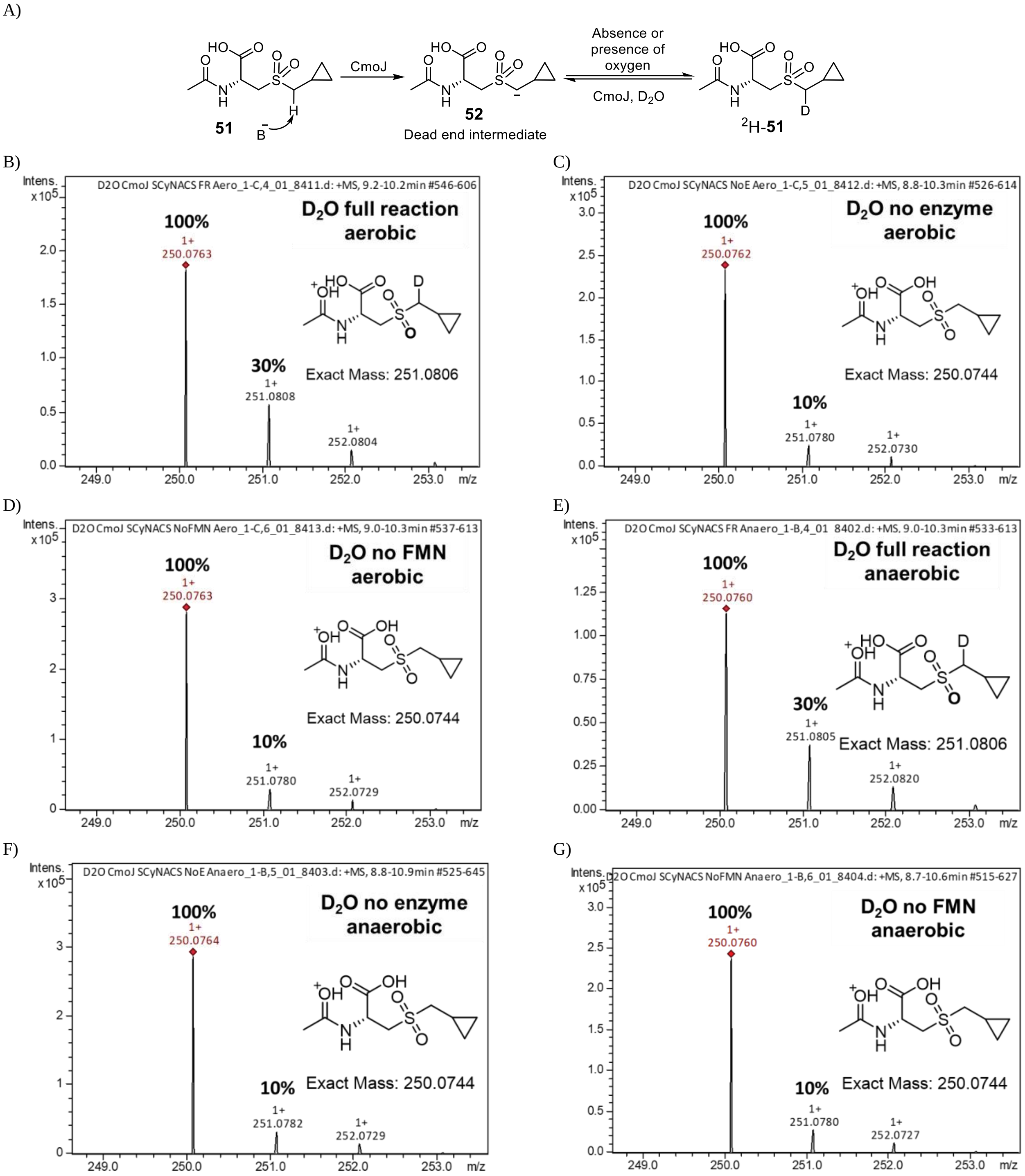

Figure S6: CmoJ-catalyzed incorporation of deuterium into N-acetyl-S-(cyclopropyl)methylcysteine sulfone **51**. A) Proposed CmoJ-catalyzed deuterium incorporation into **51**. B) CmoJ full reaction with **51** under aerobic conditions. C) Reaction with **51** in the absence of CmoJ under aerobic conditions. D) CmoJ reaction in the absence of FMN under aerobic conditions. E) CmoJ full reaction with **51** under anaerobic conditions. F) Reaction with **51** in the absence of CmoJ under anaerobic conditions. G) CmoJ reaction in the absence of FMN under anaerobic conditions. The full reaction, under aerobic and anaerobic conditions, exhibits ~17% incorporation of ^2H (Figure S4).

Derivatization of N-acetyl-L-cysteine (5) with 4,4’-dithiodipyridine (53)

In a round bottom flask, under an argon atmosphere, 0.2 mmoles of N-acetyl-L-cysteine (**5**) and 0.22 mmoles of 4,4’-dithiodipyridine-4 (**53**) were dissolved in 10 ml THF and stirred at 25 °C for 24 hr. The solvent was removed (rotovap) and the resulting crude product was dissolved in 50 mM KPi buffer pH 7.5. Disulfide **54** was purified by HPLC, characterized by NMR, and used as a reference compound to prepare a calibration curve for the kinetic studies on N-acetyl-S-(cyclopropyl)methylcysteine sulfoxide **23** described below.

A)

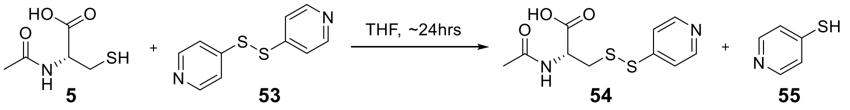

B)

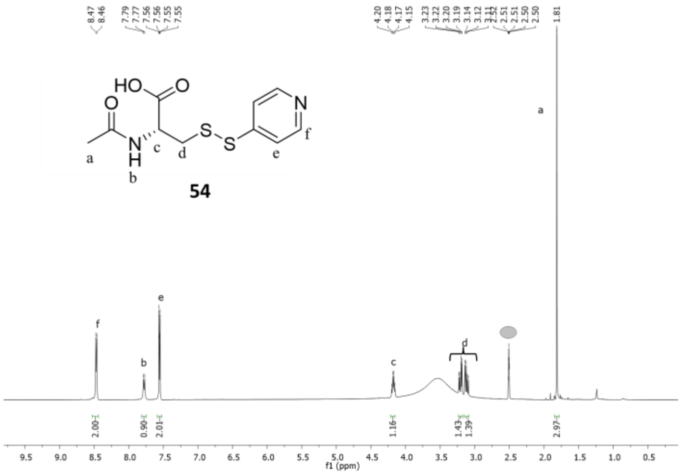

C)

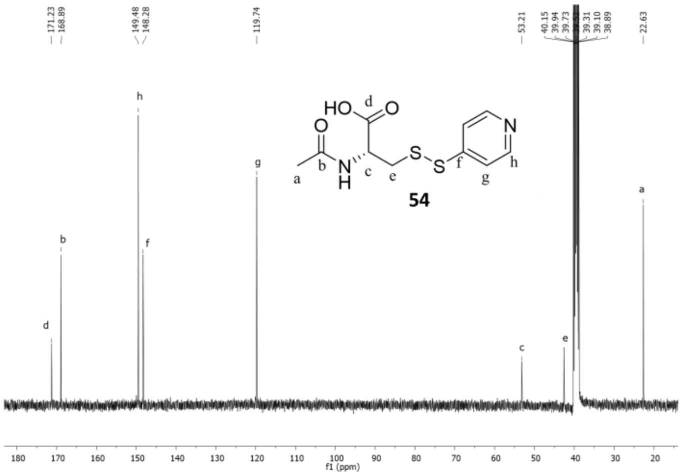

Figure S7: Synthesis and characterization of the adduct between N-acetylcysteine and 4,4’-dithiodipyridine**.** A) Synthetic scheme for the preparation of a reference sample of N-acetylcysteine-4-mercaptopyridine mixed disulfide (**54**). B) ^{1}H-NMR of **54**. C) ^{13}C-NMR of **54.**

Kinetics of CmoJ catalyzed reaction with N-acetyl-S-(cyclopropyl)methylcysteine sulfoxide

The assay mixture (100 µL) consisted of 1 µM FRE, 10-50 µM CmoJ, 50 µM FMN, 10-2000 µM substrate **23**, and 1 mM 4-mercaptopyridine[3] (**55**) in 100 mM KPi buffer, pH 7.5. The enzymatic reaction was initiated, at 25 °C, by the addition of 2 mM NADH. The reaction was quenched at suitable time points (30-600 sec) by the addition of 8 M guanidine hydrochloride (100 µL). The enzyme was removed using a 10k kDa cutoff filter and the concentration of product **54** was determined by HPLC analysis by determining the area under the signal at 254 nm. The experiments were performed in duplicates. The assay chemistry is shown in Figure S8A.

A)

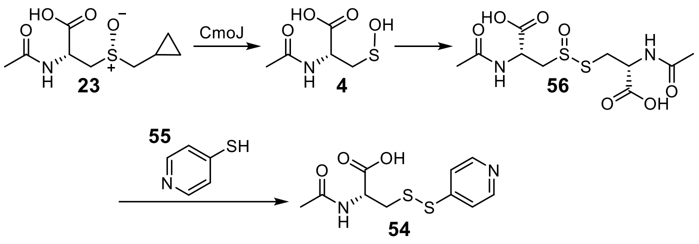

B)

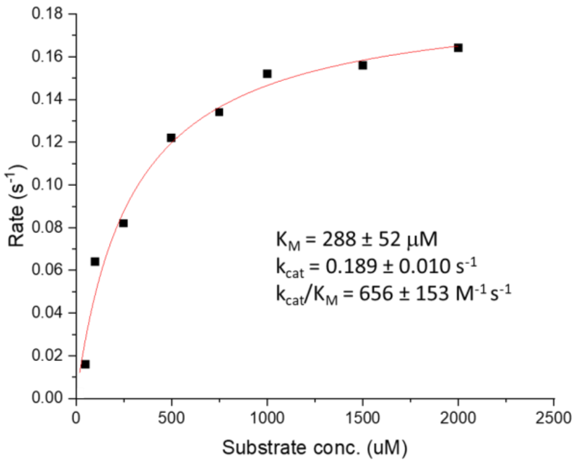

Figure S8: Michaelis-Menten kinetics of the CmoJ catalyzed reaction with N-acetyl-S-(cyclopropyl)methylcysteine sulfoxide **23**. A) Trapping of N-acetylcysteine sulfenic acid **4** and its dimer **56**, with 4-mercaptopyridine **55** to form mixed disulfide **54**; B) Michaelis-Menten plot of N-acetyl-S-(cyclopropyl)methylcysteine sulfoxide **23** with CmoJ.

Detection of cyclopropane carbaldehyde 27 formation in the CmoJ-catalyzed reaction with N-acetyl-S-(cyclopropyl)methylcysteine 23

S-(cyclopropyl)methyl-N-acetylcysteine sulfoxide **23** was generated using CmoO, as described above. The resulting reaction mixture was then used for the CmoJ reaction, without further purification. A 100 µL reaction, containing 100 µM CmoJ, 1 µM FRE, 100 µM FMN, 2 mM NADH and S-(cyclopropyl)methyl-N-acetylcysteine sulfoxide **23** solution (final conc. ~0.5 mM) in 100 mM KPi buffer pH 7.5, was incubated at 37 °C for 3 hrs. Protein was then removed

using a 10 kDa filter, and dansyl hydrazine[4] (**57**, 2 mM final conc.) was added and the reaction mixture was incubated at 60 °C for 1 hr. 40-80 µL of the sample was analyzed by LC-MS.

A)

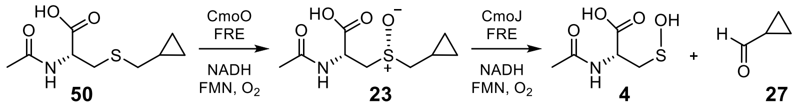

B)

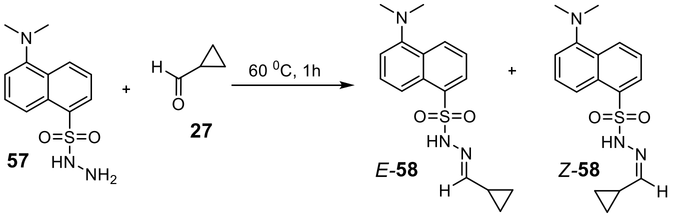

C)

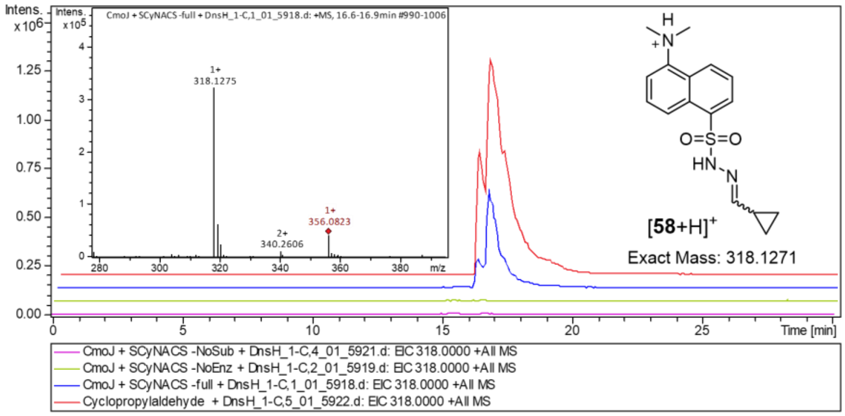

Figure S9: Evidence for the formation of cyclopropyl carbaldehyde **27**, in the CmoJ-catalyzed reaction with N-acetyl-S-(cyclopropyl)methylcysteine sulfoxide **23**. A) Sulfoxidation of N-acetyl-S-(cyclopropyl)methylcysteine sulfoxide **23** with CmoO and incubation with CmoJ forms cyclopropyl carbaldehyde **27** B) The dansyl hydrazine (**57**) derivatization reaction of cyclopropyl carbaldehyde **27** forms the *E* and *Z* stereoisomers of **58**. C) LC-MS analysis of the formation of cyclopropyl carbaldehyde **27** in the CmoJ-catalyzed reaction with N-acetyl-S-(cyclopropyl)methylcysteine sulfoxide **23** (EIC of m/z = 318 $[M+H]^+$, Red: dansyl hydrazone **58** of cyclopropane carbaldehyde, blue: dansyl hydrazine **57** treated CmoJ full reaction, green: dansyl hydrazine treated reaction mixture lacking CmoJ, pink: dansyl hydrazine treated CmoJ reaction lacking the substrate **23**. Cyclopropane carbaldehyde – dansyl hydrazone **58** was observed only in the full reaction. No other aldehydes derivatized by dansyl hydrazine were found exclusively in the full reaction.

Analysis of the CmoJ-catalyzed reaction with N-acetyl-S-(cyclopropyl)methylcysteine for vinyl sulfoxide 26

The reaction mixture consisting of 200 µM CmoJ, 1 µM FRE, 200 µM FMN, 2 mM NADH, 5 mM thiophenol, and S-(cyclopropyl)methyl-N-acetylcysteine sulfoxide **23** solution (final conc. ~0.5 mM) in 100 mM KPi buffer pH 7.5 was incubated at 37 °C for 6 hrs. Thiophenol did not inhibit CmoJ or FRE. Protein was removed using a 10 kDa cutoff filter and samples were

analyzed using HPLC. An identical reaction mixture (80 µL aliquot) was analyzed using LC-MS. No signal corresponding to the mass of **59** ([M+H]$^+$ m/z: 344.098) was detected in the EIC.

A)

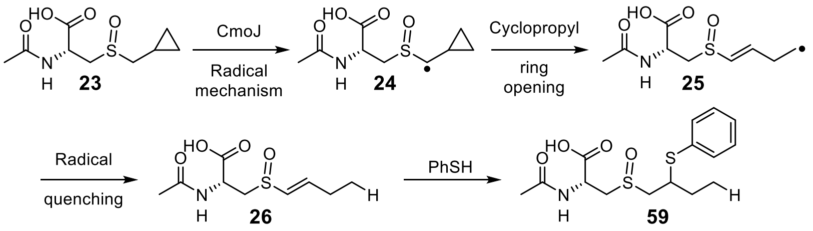

B)

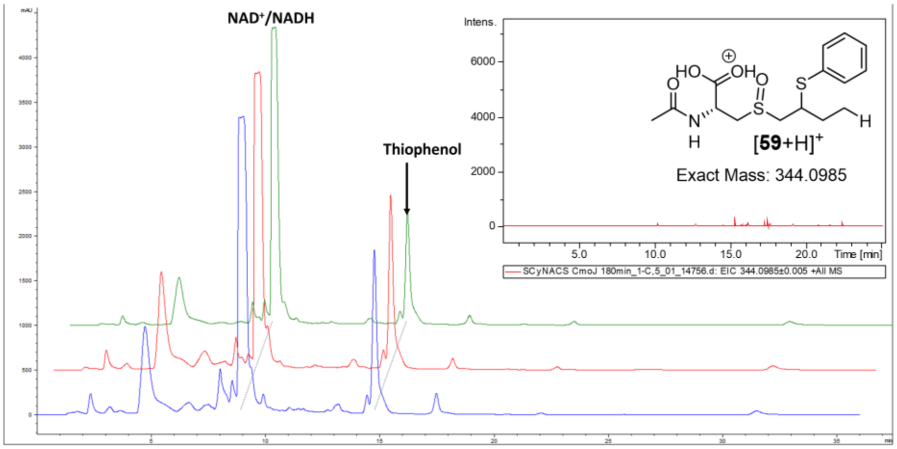

Figure S10: Strategy to detect vinyl sulfoxide **26** using thiophenol trapping. A) Proposed formation of **26** in the CmoJ-catalyzed reaction of N-acetyl-S-(cyclopropyl)methylcysteine. B) HPLC chromatogram (254 nm) of the CmoJ reaction run in the presence of thiophenol. No new peak was observed in the full reaction (Blue: full reaction + thiophenol, red: no CmoJ + thiophenol, green: no substrate + thiophenol). Inset is the EIC for **59** ([M+H]$^+$ m/z = 344.09) in the reaction mixture, and shows that **59** was not formed at detectable levels.

Sulfenic acid trapping using phenyl vinyl sulfone

A 100 µL reaction mixture, containing 100 µM CmoJ, 1 µM FRE, 100 µM FMN, 4 mM NADH, 10 mM PVSu, and 1 mM N-acetyl-S-benzyl cysteine sulfoxide **3** in 100 mM KPi-D_2O buffer pH 7.5, was incubated at 37 °C for 2 hrs. Protein was then removed using a 10 kDa filter and 90 µL of the sample was analyzed by LC-MS.

A)

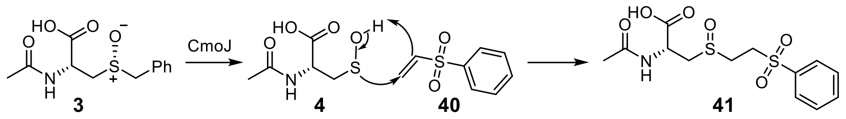

B)

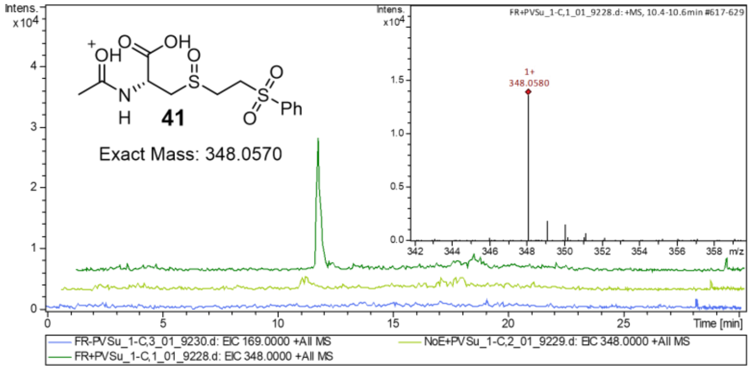

Figure S11: Strategy for trapping the sulfenic acid product **4** in the CmoJ reaction using phenyl vinyl sulfone **40**. A) Reaction between cysteine sulfenic acid (**4**) and phenyl vinyl sulfone (PVSu, **40**) to form adduct **41** B) LC-MS analysis showing the formation of adduct **41** only with the full reaction of CmoJ in the presence of PVSu (EIC of m/z = 348 $[M+H]^+$, Green: full reaction + PVSu, yellow: no CmoJ + PVSu, blue: full reaction – PVSu).

Chemoenzymatic synthesis of the adduct 41 between sulfenic acid and phenyl vinyl sulfone

10 mM N-acetylcysteine **5** and 10 mM PVSu **40** were incubated in D_2O at 60 ^oC for 12 hrs. This mixture was then analyzed for the formation of the adduct **60**, using LC-MS and NMR. Thioether **60** was then oxidized with CmoO to form sulfoxide **41**. A 100 µL reaction mixture containing 200 µM CmoO, 2 µM FRE, 200 µM FMN, 2 mM NADH, and 1 mM **60** was incubated at 37 ^oC for 6 hrs. 80 µL of the reaction mixture was analyzed by LC-MS for the formation of sulfoxide **41**. The final adduct was HPLC purified and used as a reference for the trapped sulfenic acid in the CmoJ reaction.

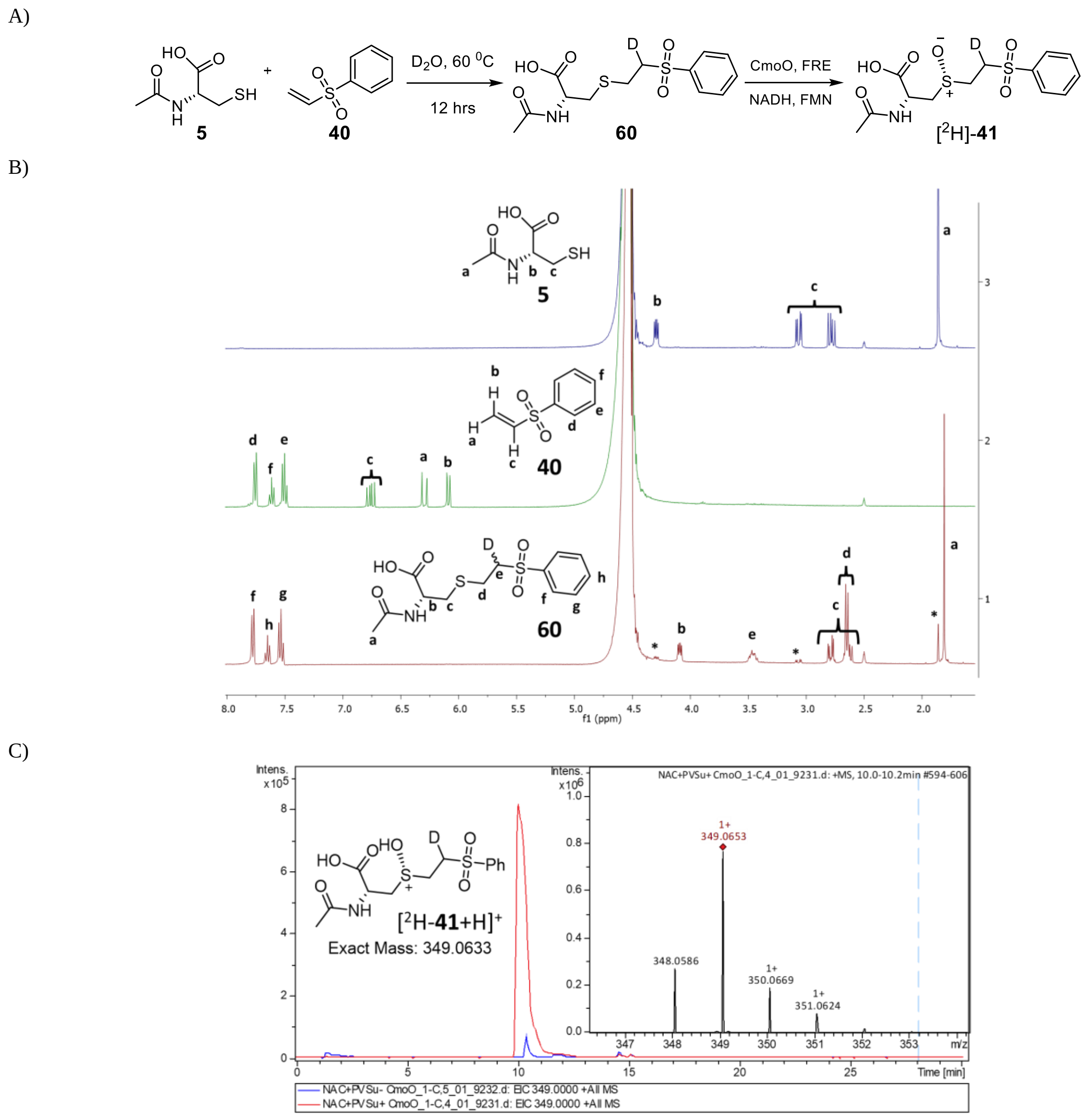

Figure S12: Synthesis of the adduct **41** between sulfenic acid **4** and phenyl vinyl sulfone **40**. A) Schematic for the synthesis of the trapped sulfenic acid **41**. B) NMR spectrum of **60**. Asterisks in panel 3 mark the peaks derived from a small amount of unreacted N-acetylcysteine C) LC-MS analysis showing the CmoO-catalyzed conversion of **60** to **41** (EIC of m/z = 349 $[M+H]^+$, Red: Complete reaction mixture, Blue: Reaction mixture with CmoO omitted. Small amounts of non-enzymatic oxidation to **41** are observed due to flavin hydroperoxide/hydrogen peroxide formation in the presence of FRE and O_2).

Synthesis of N-acetyl-S-benzylcysteine ^{18}O-sulfoxide ([^{18}O]-3) using CmoO

All buffers and reagents were transferred into the anaerobic chamber (≤ 5 ppm O_2, COY Laboratories). FRE and CmoO were buffer exchanged into anaerobic 100 mM KPi pH 7.5, using a Bio-spin 6 desalting column. Stock solutions of substrates and cofactors were also prepared in anaerobic 100 mM KPi buffer, pH 7.5.

A reaction mixture (100 µL, in an Eppendorf tube), consisting of 1 µM FRE, 100 µM CmoO, 100 µM FMN, 4 mM NADH, 2 mM N-acetyl-S-benzylcysteine **2** was made up in anaerobic 100 mM KPi pH 7.5 buffer. The Eppendorf tube was left open and placed in a round bottom flask, which was then capped with a rubber septum and sealed with Teflon tape. The sample was then removed from the anaerobic chamber and purged at least once with a $^{18}O_2$-filled balloon. The sample was then incubated with a fresh $^{18}O_2$-filled balloon. After 2 hrs, this balloon was replaced with a balloon containing Argon. The round bottom flask was subjected to a low vacuum, to replace most of the oxygen with Argon. The flask was then taken inside the glove box and opened. Protein was filtered using a 10 kDa cutoff filter and 40 µL of the sample was analyzed by LC-MS. The sulfoxide product [^{18}O]-**3** was purified by HPLC, lyophilized, and stored at -80 ^{0}C.

A)

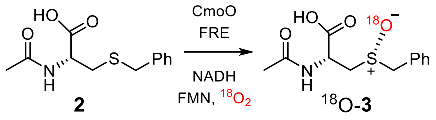

B)

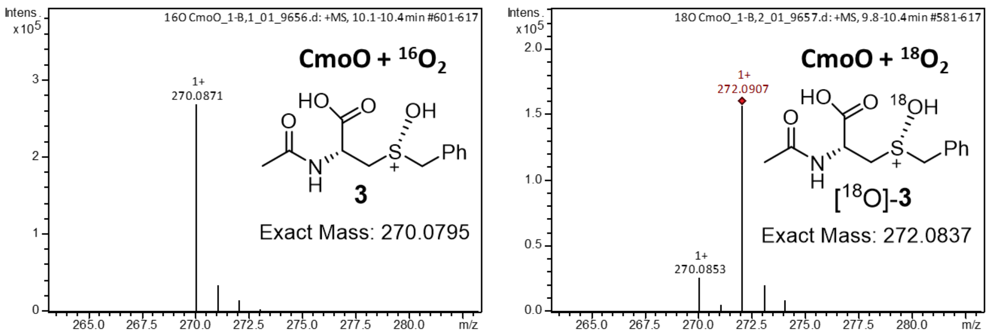

Figure S13: Synthesis of N-acetyl-S-benzylcysteine ^{18}O-sulfoxide. A) CmoO-catalyzed $^{18}O_2$-sulfoxidation of N-acetyl-S-benzylcysteine. B) LC-MS analysis of the product N-acetyl-S-benzylcysteine sulfoxide from CmoO reaction done in $^{16}O_2$ (left panel, m/z = 270) and $^{18}O_2$ (right panel, m/z = 272). The oxygen incorporation from available $^{18}O_2$ was ≅ 90%.

Determining the fate of the sulfoxide oxygen during the CmoJ reaction

The synthesized ^{18}O labeled sulfoxide substrate [^{18}O]-**3** was used to determine the fate of the sulfoxide oxygen during the CmoJ-catalyzed reaction. A 100 µL reaction mixture, consisting of 1 µM FRE, 100 µM CmoJ, 100 µM FMN, 2 mM NADH, 1 mM N-acetyl-S-benzylcysteine ^{18}O-sulfoxide ([^{18}O]-**3**), and 10 mM PVSu in 100 mM KPi pH 7.5, was incubated at 37 ^{o}C for 2 hrs. Protein was removed by ultrafiltration using a 10 kDa cutoff filter and 80 µL of the sample was analyzed by LC-MS.

Determining the source of the sulfenic acid oxygen in the CmoJ product

First, the possibility of molecular oxygen being incorporated into the sulfenic acid was investigated. All buffers and transferred to the anaerobic chamber ($\leq$ 5 ppm O_2, COY Laboratories). FRE and CmoJ were buffer exchanged into anaerobic 100 mM KPi pH 7.5 buffer using a Bio-spin 6 desalting column. Stock solutions of substrates and cofactors were also prepared in anaerobic 100 mM KPi buffer, pH 7.5 buffer.

A reaction mixture (100 µL, in an Eppendorf tube) consisting of 1 µM FRE, 100 µM CmoJ, 100 µM FMN, 2 mM NADH, 1 mM N-acetyl-S-benzylcysteine sulfoxide **3**, and 10 mM PVSu was made in anaerobic 100 mM KPi pH 7.5 buffer. The Eppendorf tube was left open and placed in a round bottom flask, which was then capped with a rubber septum and sealed with Teflon tape. The sample was then removed from the anaerobic chamber and purged at least once with a $^{18}O_2$-filled balloon. The sample was then incubated with a fresh $^{18}O_2$-filled balloon. After 2 hrs, this balloon was replaced with a balloon containing Argon. The round bottom flask was subjected to a low vacuum, to replace most of the oxygen with Argon. The flask was then opened inside the glove box. Protein was removed by ultrafiltration using a 10 kDa cutoff filter and 80 µL of the sample was analyzed by LC-MS.

Next, the possibility of incorporation of oxygen from water into the sulfenic acid was investigated. Potassium phosphate monobasic (KH_2PO_4) and dibasic (K_2HPO_4) salts were dissolved in $H_2{}^{18}O$, to obtain a 100 mM KPi pH 7.5 buffer containing ~70% $H_2{}^{18}O$. The reaction mixture was made up using this buffer as described in the previous paragraph. Analysis of the reaction mixture by LC-MS demonstrated that the trapped sulfenic acid adduct **41** had ~87% incorporation of available ^{18}O (Figure S4).

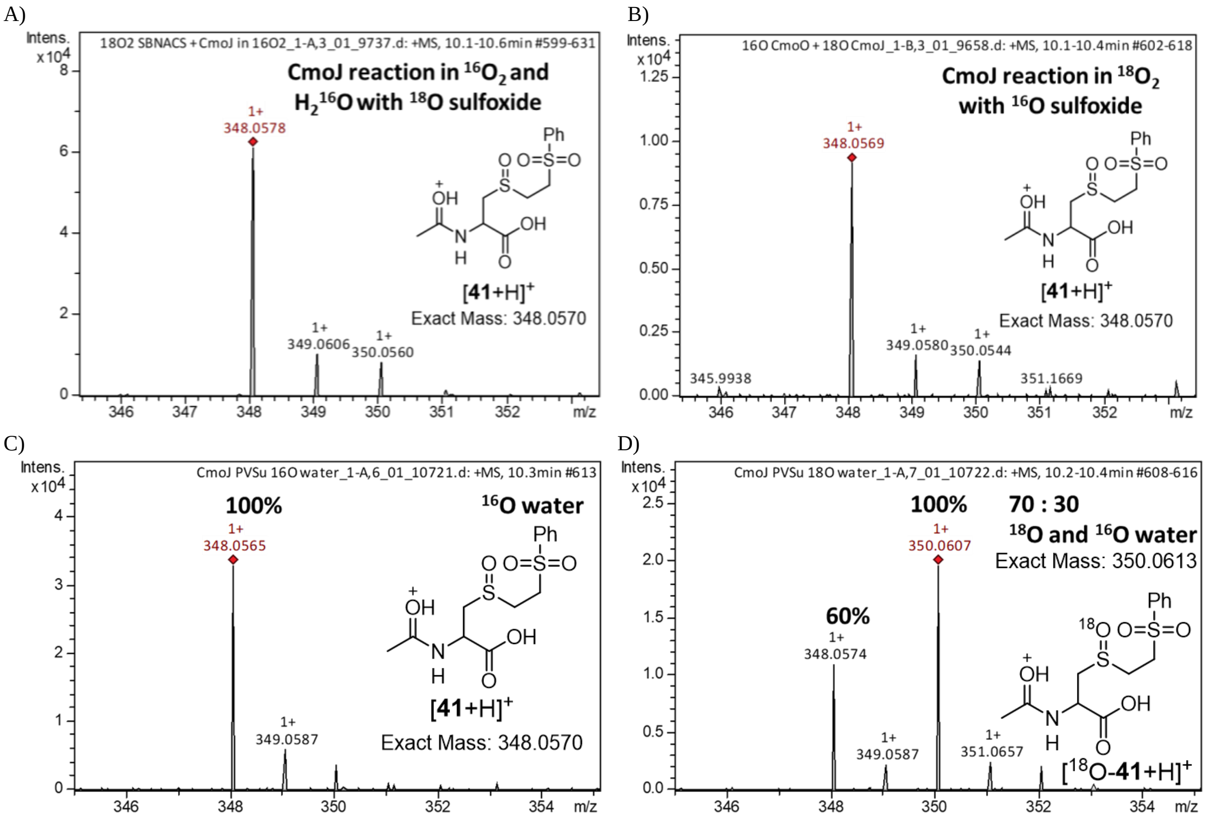

Figure S14: Determination of the origin of the sulfenic acid oxygen. A) LC-MS analysis of **41** derived from ^{18}O-labeled sulfoxide ([^{18}O]-**3**) showing that the sulfenic acid oxygen is not derived from the sulfoxide. B) LC-MS analysis of **41** derived from unlabeled sulfoxide and $^{18}O_2$ showing that the sulfenic acid oxygen is not derived from $^{18}O_2$. C) LC-MS analysis of the CmoJ reaction with **3** run in $H_2{}^{16}O$ buffer and D) CmoJ reaction run in ~70% $H_2{}^{18}O$ buffer; demonstrating that the sulfenic acid oxygen is buffer derived (~ 87% incorporation of available ^{18}O).

Model studies to probe solvent oxygen incorporation during and after adduct formation between sulfenic acid 4 and PVSu 40

Buffer oxygen incorporation into the sulfoxide of **3**: 1 mM N-acetyl-S-benzylcysteine sulfoxide **3** in 100 mM KPi - $H_2{}^{18}O$ (70%) pH 7.5 were incubated for 4 hrs at 37 ^{0}C. 40 µL of the sample was analyzed by LC-MS. No incorporation of buffer ^{18}O was observed.

Buffer oxygen incorporation into the sulfone ([^{2}H]-**41**): 1 mM sulfone ([^{2}H]-**41)** in 100 mM KPi - $H_2{}^{18}O$ (70%) pH 7.5 was incubated for 4 hrs at 37 ^{0}C. 40 µL of the sample was analyzed using LC-MS. No incorporation of buffer ^{18}O was observed.

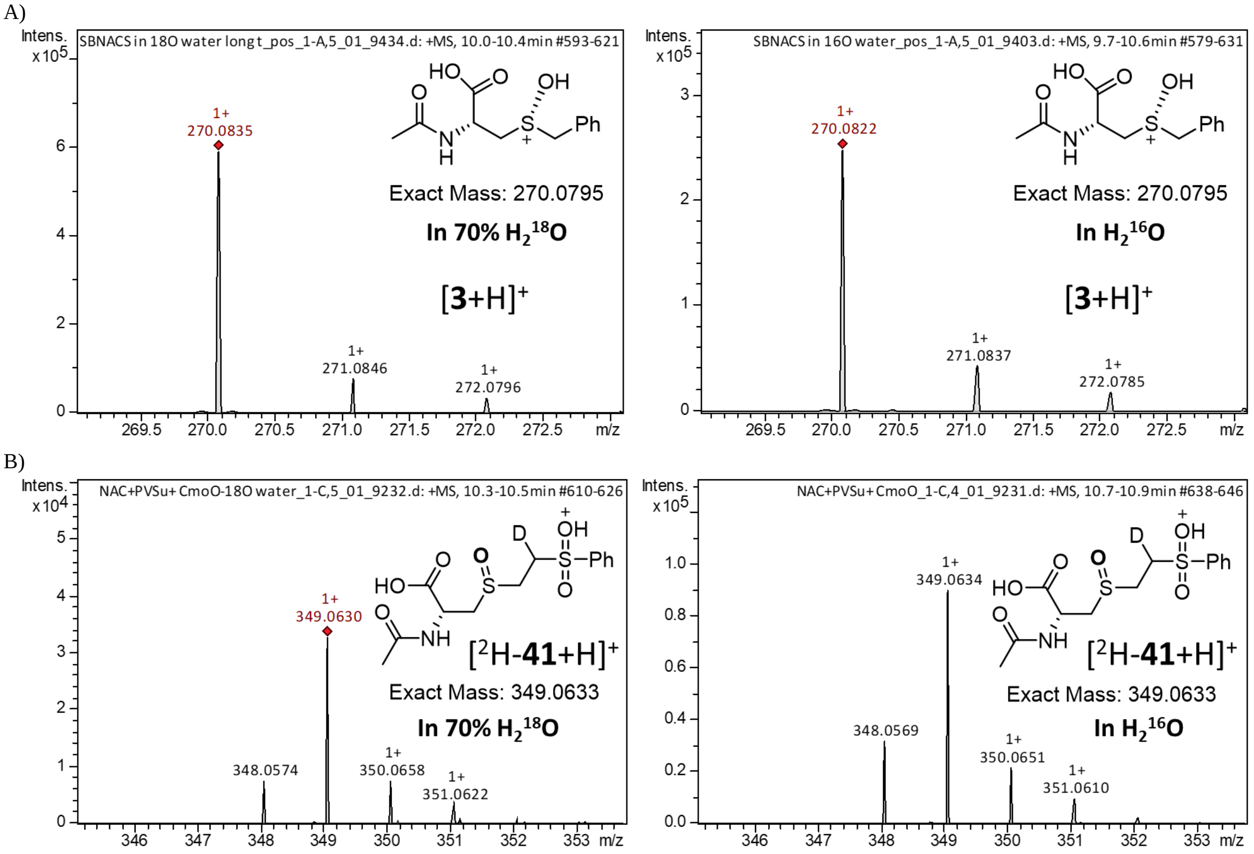

Figure S15: LC-MS studies on various possibilities for incorporation of solvent oxygen in adduct **41**. A) N-acetyl-S-benzylcysteine sulfoxide **3** incubated in $H_2{}^{18}O$ (left panel) and $H_2{}^{16}O$ (right panel). B) Standard of an adduct between N-acetylcysteine sulfenic acid and phenyl vinyl sulfone (([^{2}H]-**41**), incubated in $H_2{}^{18}O$ (left panel) and $H_2{}^{16}O$ (right panel).

Synthesis of photocaged N-acetylcysteine sulfenic acid precursor 42

A photocaged N-acetylcysteine sulfenic acid was synthesized by modifying the reported procedures, as described below.[5]

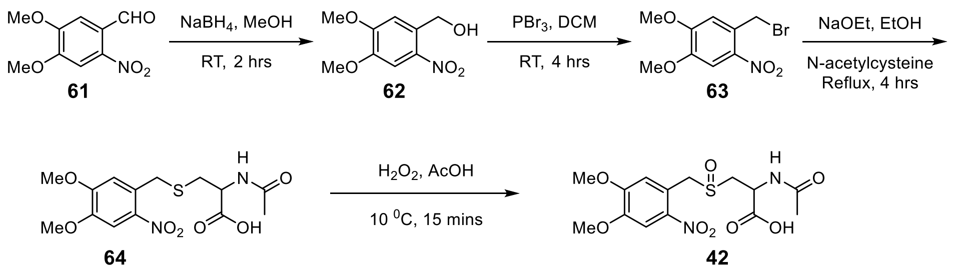

Figure S16: Synthesis of N-acetyl-S-(4,5-dimethoxy-2-nitrobenzyl)-L-cysteine sulfoxide **42**, as a photocaged precursor to N-acetylcysteine sulfenic acid **4**.

6-nitroveratrole 62: Commercially available 6-nitroveratraldehyde (**61**, 2.1 g) was dissolved in 50 mL methanol and cooled to 0 ^oC. Then $NaBH_4$ (190 mg, 0.5 eq.) was added portion-wise over 5 min. The reaction was stirred at room temperature for 2 hrs. The reaction was quenched with 1 N aqueous NH_4Cl (10 mL) and most of the methanol was evaporated. The aqueous fraction was then extracted with ethyl acetate (2 x 50 mL). Organic fractions were pooled and washed with brine (20 mL), dried over Na_2SO_4, and evaporated to yield a yellow solid **62** (yield: 98%). The solid was used without further purification. ^{1}H NMR (400 MHz, DMSO) δ 7.65 (s, 1H), 7.38 (s, 1H), 5.55 (t, *J* = 5.4 Hz, 1H), 4.82 (d, *J* = 5.3 Hz, 2H), 3.90 (s, 3H), 3.85 (s, 3H).

1-(bromomethyl)-4,5-dimethoxy-2-nitrobenzene 63: 6-nitroveratrole (**62**, 2.1 g) was dissolved in 30 mL DCM and cooled to 0 ^oC. PBr_3 (0.95 mL, 1 eq.) was added, and the reaction mixture was stirred at room temperature for 4 hrs and quenched with 0.5 M NaOH (50 mL). The aqueous layer was extracted with DCM (2 x 50 mL) and all organic fractions were pooled. The organic fractions were washed with brine (40 mL), dried over Na_2SO_4, and evaporated. The bright yellow solid obtained (yield 85%) was used without further purification. ^{1}H NMR (400 MHz, $CDCl_3$) δ 7.66 (s, 1H), 6.94 (s, 1H), 4.86 (s, 2H), 3.99 (s, 3H), 3.95 (s, 3H).

N-acetyl-S-(4,5-dimethoxy-2-nitrobenzyl)-L-cysteine 64: In a 50 mL round-bottomed flask, N-acetylcysteine (**5**, 163 mg, 1 mmol) was dissolved in 10 mL anhydrous ethanol, under an Argon atmosphere. The solution was stirred for 5 mins at room temperature and sodium ethoxide (21% in ethanol, 0.72 mL, 2.2 eq.) was added. The solution was stirred for 15 mins at room temperature, under an Argon atmosphere. Then a solution of 1-(bromomethyl)-4,5-dimethoxy-2-nitrobenzene (**63**, 275 mg, 1 eq.) in 5 mL anhydrous acetonitrile was added. The reaction was refluxed for 4 hrs., then cooled to room temperature and quenched with 2 mL water. Most of the ethanol was evaporated and 1 N HCl (10 mL) was added to the residue. The residue was extracted with ethyl acetate (3 x 20 mL) and all organic fractions were pooled. The organic fractions were washed with brine, dried with Na_2SO_4, and evaporated. A sticky residue was obtained, from which the product

was recrystallized using acetone, ether, and methanol (yield 58%). ^{1}H NMR (400 MHz, DMSO) δ 8.20 (d, *J* = 7.9 Hz, 1H), 7.66 (s, 1H), 7.13 (s, 1H), 4.38 (td, *J* = 8.1, 5.2 Hz, 1H), 4.07 (q, *J* = 13.6 Hz, 2H), 3.91 (s, 3H), 3.86 (s, 3H), 2.83 (dd, *J* = 13.8, 5.1 Hz, 1H), 2.68 (dd, *J* = 13.8, 8.4 Hz, 1H), 1.85 (s, 3H).

N-acetyl-S-(4,5-dimethoxy-2-nitrobenzyl)-L-cysteine sulfoxide 42: In a 10 mL round-bottomed flask, N-acetyl-S-(4,5-dimethoxy-2-nitrobenzyl)-L-cysteine (**64**, 15 mg, 0.04 mmol) was dissolved in 2 mL acetic acid. The reaction was cooled to 10 ^{o}C and H_2O_2 (15 μL of 33% w/v) was added. The reaction was stirred at 10 ^{o}C for 15 mins and then acetic acid was removed at 0 ^{o}C (avoid heating up to reduce the risk of overoxidation) by a stream of air. The residue was washed with diethyl ether (2 x 10 mL) to obtain a light brown solid. This method of making the sulfoxide produces a mixture of diastereomers, as the reaction is not stereoselective. ^{1}H NMR (400 MHz, DMSO) δ 8.44 (d, *J* = 8.0 Hz, 1H), 8.38 (d, *J* = 8.0 Hz, 1H), 7.69 (d, *J* = 9.2 Hz, 2H), 7.19 (d, *J* = 15.1 Hz, 2H), 4.70 (dd, *J* = 17.5, 12.5 Hz, 2H), 4.59 (dd, *J* = 14.1, 7.3 Hz, 1H), 4.47 (ddd, *J* = 11.4, 8.0, 3.6 Hz, 1H), 4.36 (ddd, *J* = 22.2, 12.9, 8.8 Hz, 2H), 3.90 (d, *J* = 2.4 Hz, 6H), 3.88 (d, *J* = 1.8 Hz, 6H), 3.17 – 3.04 (m, 2H), 2.93 (dd, *J* = 13.3, 7.4 Hz, 2H), 1.86 (s, 3H), 1.84 (s, 3H).

Photo-generation of N-acetylcysteine sulfenic acid and its *in situ* trapping with phenyl vinyl sulfone

For the photochemical reactions, a uvBeast™ 365 nm LED light was used. A typical reaction mixture contained 1 mM sulfoxide **42** in 100 mM KPi pH 7.5. The reaction was carried out in an Eppendorf tube that was covered with aluminum foil with the reflective surface on the inside. The cap of the tube was left open, and the light was set up from the top, to focus the beam on the lower tip of the Eppendorf tube. The solution was illuminated for 2 hrs. at 37 ^{o}C and analyzed for substrate consumption by HPLC.

To trap the sulfenic acid product, PVSu (**40**) was used at the same concentration used to trap the sulfenic acid in the enzymatic reaction. A typical reaction mixture contained 1 mM of sulfoxide **42** and 10 mM PVSu in 100 mM KPi pH 7.5. The solution was illuminated for 2 hrs. at 37 ^{o}C as described above and analyzed for the trapped product **41** by LC-MS.

An identical reaction carried out in 70% $H_2{}^{18}O$-KPi buffer (100 mM pH ~7.5) with 1 mM sulfoxide **42** and 10 mM PVSu did not show any incorporation of ^{18}O into the trapped sulfenic acid **41**. However, lowering the concentration of PVSu to 2 mM, while keeping the substrate **42** at 1 mM resulted in ~60% incorporation of the available ^{18}O into the trapped sulfenic acid.

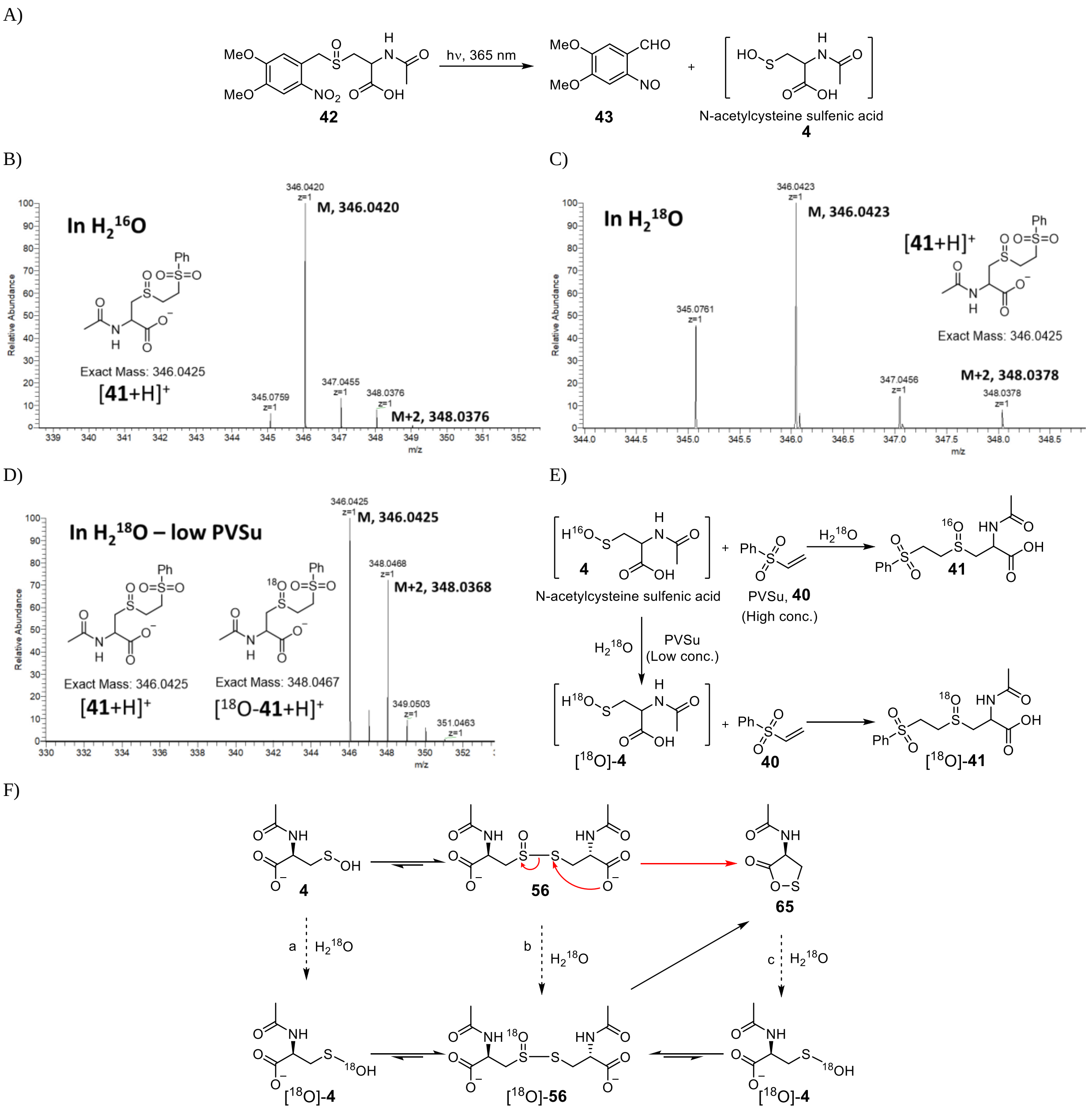

Figure S17: Evaluation of oxygen exchange in photogenerated N-acetylcysteine sulfenic acid. A) Scheme for the light-mediated synthesis of N-acetylcysteine sulfenic acid;[5] B) LC-MS trace showing the isotopic distribution of adduct **41** from photo-generated N-acetylcysteine sulfenic acid and PVSu incubated in $H_2{}^{16}O$-buffer ([**42**] = 1mM and [PVSu] = 10 mM). C) LC-MS trace showing the isotopic distribution of adduct **41** from photo-generated N-acetylcysteine sulfenic acid and PVSu incubated in 70% $H_2{}^{18}O$-buffer ([**42**] = 1mM and [PVSu] = 10 mM) showing no incorporation of ^{18}O; D) LC-MS trace showing the isotopic distribution of adduct **41** from photo-generated N-acetylcysteine sulfenic acid and PVSu incubated in 70% $H_2{}^{18}O$-buffer ([**42**] = 1mM and [PVSu] = 2 mM) showing ~55% incorporation of available ^{18}O; E) A schematic showing that oxygen exchange competes with trapping at low concentrations of PVSu, F) Possible routes for the incorporation of solvent ^{18}O into N-acetylcysteine sulfenic acid **4**.

Table S1: Effect of trapping agent concentration on the exchange of N-acetylcysteine sulfenic acid **4** with buffer.[a]

[PVSu]	[Sulfenic acid precursor 42]	Incorporation of available ^{18}O
2 mM	1 mM	63%
5 mM	1 mM	30%
10 mM	1 mM	<5%
25 mM	1 mM	<2%

[a.]The level of incorporation was calculated based on the formula derived from Figure S4.

Evaluation of sulfenic acid oxygen exchange during the CmoJ-catalyzed reaction

To determine the origin of the sulfenic acid oxygen in the CmoJ product, it was necessary to compare the extent of oxygen exchange, in sulfenic acid generated under similar conditions, in the enzymatic and photochemical systems. This was accomplished by running reactions in which PVSu was in excess (10 mM), substrate concentrations [**3** (enzymatic)] **=** [**42** (photochemical)] = 1 mM, and by matching the light intensity and the enzyme concentrations to equalize the rates of cysteine sulfenic acid production.

Photochemical generation of the sulfenic acid **4**: A typical reaction mixture contained 1 mM of sulfoxide **42** in 100 mM KPi pH 7.5 in a foil-covered Eppendorf tube as described above. The reaction was illuminated through a 0.3 OD filter (Edmund Optics stock #46-212, transmittance = 0.5). Samples were collected every 5 mins and analyzed for substrate consumption by HPLC (Figure S18).

CmoJ-catalyzed formation of the sulfenic acid **4**: A typical reaction mixture, containing 100 µM CmoJ, 1 µM FRE, 100 µM FMN, 4 mM NADH, 2 mM 4-mercaptopyridine, and 1 mM N-acetyl-S-benzyl cysteine sulfoxide **3** in 100 mM KPi buffer pH 7.5, was incubated at 37^0C. Samples were collected every 5 mins and analyzed for the 4-mercaptopyridine trapped sulfenic acid **54** by HPLC (Figure S18).

To determine the amount of solvent oxygen incorporation in PVSu-trapped sulfenic acid, a typical photochemical reaction contained 1 mM of sulfoxide **42** and 10 mM PVSu in 50% $H_2{}^{18}O$-100 mM KPi pH 7.5 in a foil-shielded Eppendorf tube and illuminated through the 0.3 OD filter. For the corresponding enzymatic reaction, a reaction mixture containing 100 µM CmoJ, 1 µM FRE, 100 µM FMN, 4 mM NADH, 10 mM PVSu, and 1 mM N-acetyl-S-benzyl cysteine sulfoxide **3** in 50% $H_2{}^{18}O$-KPi 100 mM pH 7.5 was incubated at 25 ^{0}C. Samples were collected every 5 minutes from each of the reactions and analyzed by LC-MS (Figure S18).

A)

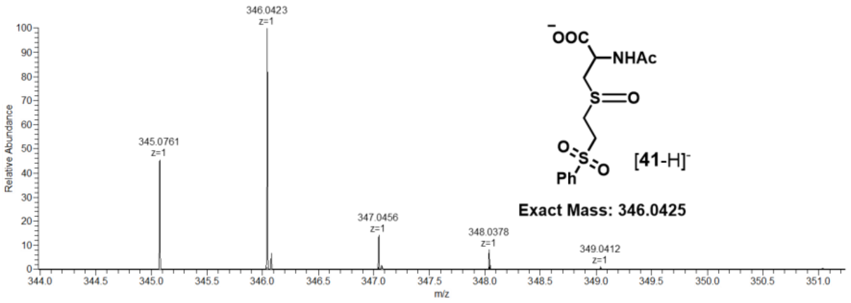

B)

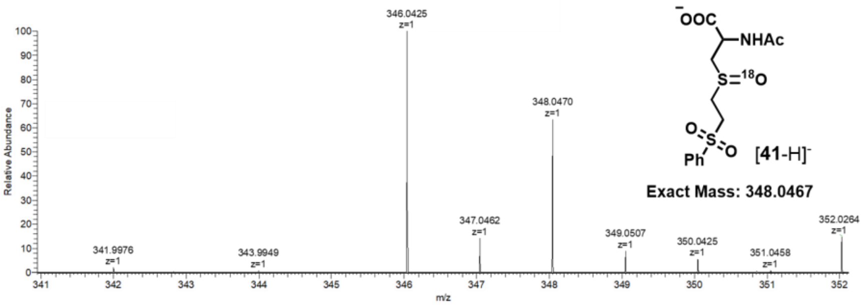

C)

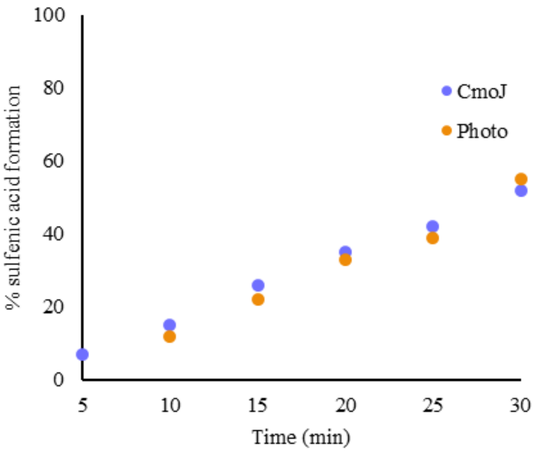

D)

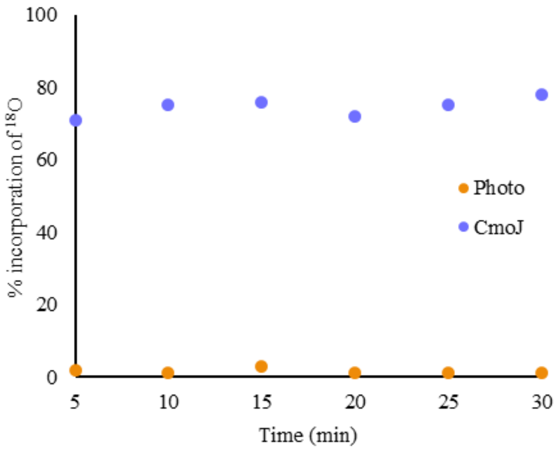

Figure S18: Incorporation of oxygen from water into the photochemically and enzymatically produced sulfenic acid **4**. A) LC-MS of the trapped photogenerated sulfenic acid **41**, after 50% conversion, showing no incorporation of ^{18}O; B) LC-MS of the enzymatically generated sulfenic acid **41**, after 50% conversion, showing ~80% incorporation of available ^{18}O; C) A plot of sulfenic acid formed enzymatically (blue circles) and photochemically (orange circles) from 1 mM substrate, D) A plot of the amount of ^{18}O incorporated into sulfenic acid formed enzymatically (blue circles) and photochemically (orange circles) from 1 mM substrate **3** (enzymatic) and **42** (photochemical).

Trapping of an FMN N5-peroxide intermediate[6] in the CmoJ-catalyzed reaction

All buffers and reagents were transferred into the anaerobic chamber ($\leq$ 5 ppm O_2, COY Laboratories). CmoJ was buffer exchanged into anaerobic 100 mM KPi pH 7.5 buffer, using a Bio-spin 6 desalting column.

A 100 µL mixture containing 0.5 mM FMN and 10 mM EDTA in H_2O was photo-reduced by irradiation with a 100 W white light LED bulb, till the color of the solution disappeared (5-10 mins). This solution was used in the CmoJ reaction mixture. A 100 µL reaction mixture containing 150 µM CmoJ, photo-reduced FMN (final conc. of reduced FMN ~100 µM), and 2 mM N-acetyl-S-benzylcysteine in 100 mM KPi pH 7.5, was incubated at 37 °C for 10 mins inside the anaerobic chamber. Then it was taken out and exposed to atmospheric oxygen, for 15 mins with shaking at 120 rpm. Protein was removed by ultrafiltered using a 10 kDa cutoff filter and 90 µL of the sample was analyzed by LC-MS

A)

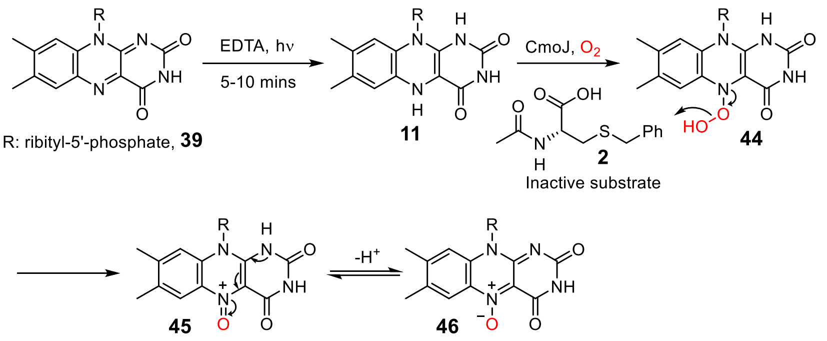

B)

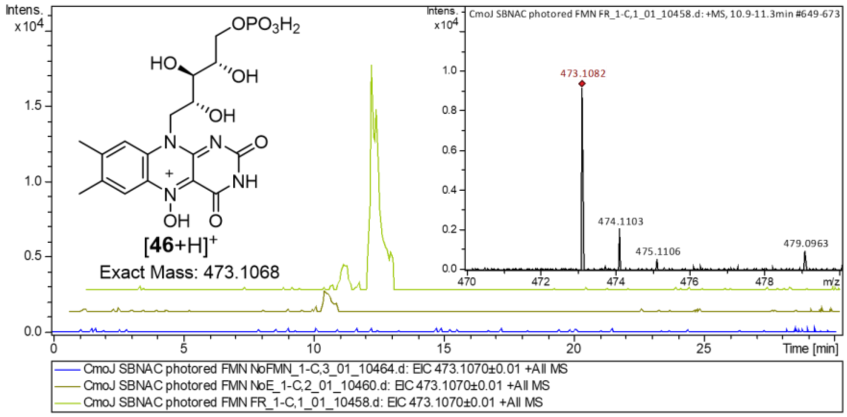

Figure S19: Trapping FMN N5-peroxide intermediate **46**[6] in the CmoJ-catalyzed reaction. A) Trapping of the FMN N5-peroxide intermediate, as a stable FMN N5-oxide, when the CmoJ reaction is carried out in the presence of an inactive substrate analog **2**, B) LC-MS analysis showing the formation of FMN N5 oxide **46** only in the presence of photo-reduced FMN, substrate analog **2**, and CmoJ (EIC of m/z = 473 $[M+H]^+$, Green: full reaction, brown: no substrate analog, blue: no enzyme). Insert shows the MS of the FMN N5 oxide formed in the reaction mixture.

References

(1) Biemann, K. *Mass spectrometry: organic chemical applications*; McGraw-Hill, 1962.

(2) Mikkelsen, K.; Nielsen, S. O. Acidity measurements with the glass electrode in H2O-D2O mixtures. *J. Phys. Chem.* **1960**, *64* (5), 632-637.

(3) Gupta, V.; Carroll, K. S. Sulfenic acid chemistry, detection and cellular lifetime. *Biochim. Biophys. Acta - Gen. Subj.* **2014**, *1840* (2), 847-875.

(4) Chayen, R.; Dvir, R.; Gould, S.; Harell, A. 1-Dimethylaminonaphthalene-5-sulfonyl hydrazine (dansyl hydrazine): a fluorometric reagent for carbonyl compounds. *Anal. Biochem.* **1971**, *42* (1), 283-286.

(5) Pan, J.; Carroll, K. S. Light-Mediated Sulfenic Acid Generation from Photocaged Cysteine Sulfoxide. *Org. Lett.* **2015**, *17* (24), 6014-6017.

(6) Matthews, A.; Saleem-Batcha, R.; Sanders, J. N.; Stull, F.; Houk, K.; Teufel, R. Aminoperoxide adducts expand the catalytic repertoire of flavin monooxygenases. *Nat. Chem. Biol.* **2020**, *16* (5), 556-563.
